# ANAC102 predominantly expresses a nuclear protein and acts as a negative regulator of methyl viologen-induced retrograde signaling

**DOI:** 10.1093/jxb/erae235

**Published:** 2024-05-30

**Authors:** Xiaopeng Luo, Xinqiang Jiang, Vivian Schmitt, Shubhada R Kulkarni, Huy Cuong Tran, Sylwia M Kacprzak, Frank Van Breusegem, Olivier Van Aken, Klaas Vandepoele, Inge De Clercq

**Affiliations:** 1 Ghent University, Department of Plant Biotechnology and Bioinformatics, 9052 Ghent, Belgium; 2 VIB Center for Plant Systems Biology, 9052 Ghent, Belgium; 3 Department of Biology, Lund University, Lund 223 62, Sweden; 4 VIB Center for AI & Computational Biology, VIB, Ghent, Belgium; ‡ College of Landscape Architecture and Forestry, Qingdao Agricultural University, Qingdao, 266109, Shandong, China; † European Center for Angioscience, Medical Faculty Mannheim, Heidelberg University, Heidelberg, Germany; Division of Vascular Oncology and Metastasis, German Cancer Research Center Heidelberg (DKFZ-ZMBH Alliance), Heidelberg, Germany

**Keywords:** *Arabidopsis*, transcription factors, retrograde signaling, chloroplasts, oxidative stress, gene regulatory networks

## Abstract

Plants, being sessile organisms, constantly need to respond to environmental stresses, often leading to the accumulation of reactive oxygen species (ROS). While ROS can be harmful, they also act as messengers guiding plant growth and stress responses. Because chloroplasts are sensitive to environmental changes and are both a source and target of ROS during stress conditions, they are important in conveying environmental changes to the nucleus, where acclimation responses are coordinated to maintain organellar and overall cellular homeostasis. ANAC102 has previously been established as a regulator of β-cyclocitral-mediated chloroplast-to-nucleus signaling, protecting plants against photooxidative stress. However, debates persist about where ANAC102 is located – in chloroplasts or in the nucleus. Our study, utilizing the genomic *ANAC102* sequence driven by its native promoter, establishes ANAC102 primarily as a nuclear protein, lacking a complete N-terminal chloroplast-targeting peptide. Moreover, our research reveals the sensitivity of plants overexpressing *ANAC102* to severe superoxide-induced chloroplast oxidative stress. Transcriptome analysis unraveled ANAC102's dual role in negatively and positively regulating genome-wide transcriptional responses to chloroplast oxidative stress. Through the integration of published data and our own study, we constructed a comprehensive transcriptional network, which suggests that ANAC102 exerts direct and indirect control over transcriptional responses through downstream transcription factor networks, providing deeper insights into the ANAC102-mediated regulatory landscape during oxidative stress.

## Introduction

Due to their sedentary nature, plants must continuously acclimate to changing environmental conditions, such as temperature shifts, water availability and high light levels, which often lead to the accumulation of reactive oxygen species (ROS) within the plant. Excessive ROS accumulation can disrupt redox homeostasis and causes progressive oxidative damage, ultimately leading to cell death. Conversely, ROS can serve as signaling molecules, acting as second messengers extensively coordinating plant growth, development, and acclimation in response to environmental as well as intracellular changes (Gechev *et al*., 2006; Baxter *et al*., 2014; Considine and Foyer, 2021; Mittler *et al*., 2022). During stresses, ROS production is increased in organelles due to perturbation of their metabolic processes such as photosynthesis, photorespiration and/or respiration. To counteract oxidative stress and to maintain cellular energy homeostasis, activities of the organelles are tightly coordinated with the transcriptional machinery in the nucleus. This involves both anterograde regulation (signaling from the nucleus to organelles) and retrograde signaling (communication from organelles to the nucleus) (Woodson and Chory, 2008).

**Fig. 1:**
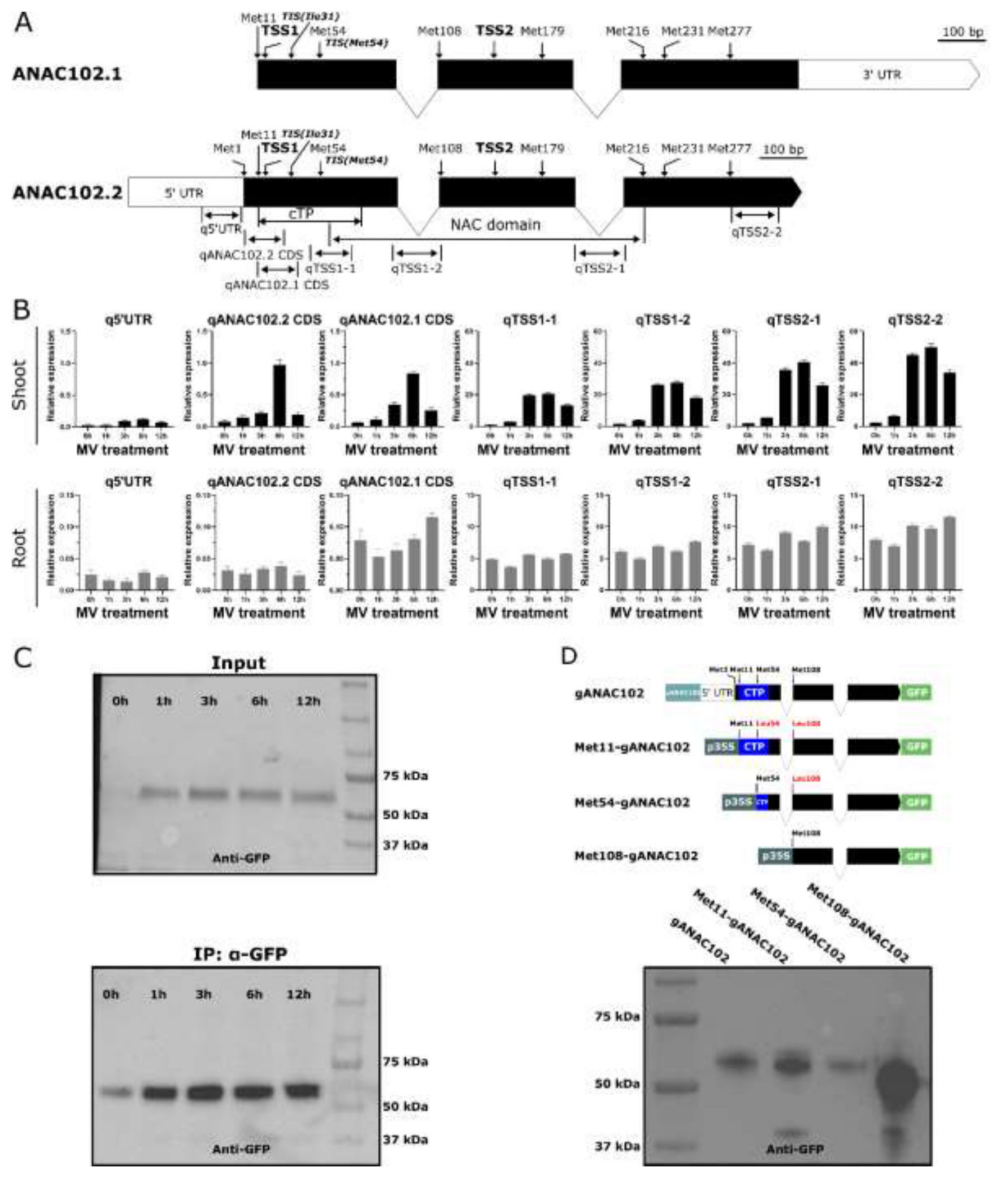
*ANAC102* predominantly expresses a nuclear protein isoform. (A) Overview of RT-qPCR the *ANAC102* gene model with transcription start sites (TSS1 and TSS2, identified by Ushijima *et al*. (2017) and (Nielsen *et al*., 2019), in-frame AUG start codons (Met), and translation initiation sites (TIS, identified by Willems *et al*. (2022) indicated in the *ANAC102.1 and ANAC102.2* gene models (according to TAIR; arabidopsis.org). cTP, chloroplast-targeting peptide (80-amino-acid sequence, Xin *et al*., 2021)); q, RT-qPCR amplicon corresponding to the 5’UTR, *ANAC102.2* coding sequence (CDS), the *ANAC102.1* CDS, and the TSS1 and TSS2 transcript models, respectively. (B) RT-qPCR analysis of the different *ANAC102* transcript models in *Arabidopsis thaliana* wild-type seedlings during an MV stress time series. Error bars represent the standard deviation (n = 4 biological replicates). (C) Western blot analysis of the ANAC102 protein model in total protein extracts, before (input) and after anti-GFP immunoprecipitation (IP), from *Arabidopsis* seedlings stably expressing p*ANAC102::*g*ANAC102-GFP* during an MV stress time series. (D) Western blot analysis of the genomic *ANAC102* gene fragment [-1500 +1395] fused to GFP (p*ANAC102::*g*ANAC102-GFP*) after transient expression in *N. benthamiana* and comparison with different N-terminally truncated protein isoforms initiated at different in-frame AUG start codons (Met11, Met54 and Met108) and with the downstream start codon(s) (Met54 and Met108) mutated.

Due to the sensitivity of chloroplastic processes to various stresses, chloroplasts are thought to serve as crucial sensors and intermediaries between environmental fluctuations and responses in the nucleus (Crawford *et al*., 2018; Schwenkert *et al*., 2022). Stress-triggered chloroplast retrograde signals are mediated by ROS, metabolites including β-cyclocitral (β-cc), 2-C-methyl-d-erythritol 2,4-cyclodiphosphate, 3ʹ-phosphoadenosine 5ʹ-phosphate, and intermediates of the tetrapyrrole biosynthesis pathway (Moulin *et al*., 2008; Estavillo *et al*., 2011; Ramel *et al*., 2012; Xiao *et al*., 2012; Terry and Smith, 2013; Dietz *et al*., 2016; Exposito-Rodriguez *et al*., 2017). ANAC102 has been identified as a key player in β-cc-mediated chloroplast retrograde signaling, positioned upstream of at least three other NO APICAL MERISTEM/ARABIDOPSIS TRANSCRIPTION ACTIVATION FACTOR/CUP-SHAPED COTYLEDON (NAC) transcription factors (TFs) (ANAC002, ANAC032 and ANAC081), inducing a detoxification response and facilitating plant acclimation to photooxidative stress (D’Alessandro *et al*., 2018). Simultaneously, ANAC102 has been implicated in cadmium (Cd) tolerance (Han *et al*., 2023), in plant growth during mild methyl viologen-induced oxidative stress and in protecting germinating seeds to low oxygen stress (Christianson *et al*., 2009; De Clercq *et al*., 2021). Despite its essential functions, there is an ongoing debate regarding the subcellular localization of ANAC102. Initial reports, based on the expression of the annotated coding sequence (*ANAC102.1*) from the Cauliflower Mosaic Virus 35S promoter (p35S), in both *Arabidopsis thaliana* and *Nicotiana benthamiana*, suggested that ANAC102 is a chloroplast-localized TF (Inzé *et al*., 2012). Subsequent research contended that ANAC102 is present in both the nucleus and chloroplasts, in the latter compartment interacting with RNA polymerases to regulate the transcription of chloroplast genes (Xin *et al*., 2021). Recent findings reported a nuclear localization and function for ANAC102 by binding to the promoter and regulating expression of a gene involved in Cd responses (Han *et al*., 2023). Moreover, ANAC102 was observed to exclusively localize in the nucleus when expressed without the predicted N-terminal chloroplast-targeting peptide (cTP), which was found to be sufficient for chloroplast targeting (Inzé, 2012; Xin *et al*., 2021). Adding to the complexity, ANAC102 has been reported to possess two transcription start sites (TSSs) that are alternatively selected during red light exposure (Ushijima *et al*., 2017). While the two mRNA models according to TAIR10 (*ANAC102.1* and *ANAC102.2*; arabidopsis.org; Fig. 1A) include the cTP in the coding sequence, both TSSs identified by Ushijima *et al*. (2017), respectively positioned within and downstream of the cTP, exclude expression of the complete cTP.

A complete view on the different TSSs and whether the corresponding protein model(s) include the (complete) cTP, remain unclear. Therefore, we investigated the subcellular localization of ANAC102 using its genomic sequence controlled by its native promoter. Our findings revealed that *ANAC102* predominantly expresses a nuclear protein isoform, omitting the N-terminal extension containing the cTP. Furthermore, our observations indicate that ANAC102 serves as a positive and negative regulator of methyl viologen-induced chloroplast retrograde signaling in the nucleus, both directly through transcriptional regulation of target genes and indirectly through transcription factor networks.

## Materials and methods

### Plant material and growth conditions

*A. thaliana* seedlings were grown at 21°C under a 16-h light/8-h dark photoperiod on half-strength Murashige and Skoog (½ MS) medium (Duchefa Biochemie, Haarlem, The Netherlands), supplemented with 1% (w/v) sucrose, 0.75% (w/v) agar, and adjusted to pH 5.7, following stratification for 48 h. The *Arabidopsis* lines overexpressing (OE) the full-length *ANAC102.1* (AT5G63790, TAIR, Arabidopsis.org)-coding sequence from the p35S in Col-0 background have been described previously (Inzé, 2012). The T-DNA insertion lines for *ANAC102* (SALK_030702C; *anac102* knockout [KO]) were obtained from the European Arabidopsis Stock Centre. The homozygous T-DNA insertion line was confirmed by PCR with gene-specific primers (LP and RP) and a T-DNA-specific primer (LB; Supplementary Table S1). Quantitative real-time PCR (RT-qPCR) analysis indicated 1% residual ANAC102 mRNA levels compared to the wild type (WT). The translational fusion constructs were obtained using Gateway cloning (Karimi *et al*., 2002). The 2895-bp genomic region comprising the 5’ upstream regulatory region and genomic DNA of *ANAC102* [-1500 +1395] was amplified from *Arabidopsis* Col-0 genomic DNA using iProof high-fidelity DNA polymerase (Bio-Rad) and cloned into the pB7FWG destination vector (primers used are shown in Supplementary Table S1). The synthetic N-terminal truncated *ANAC102* gene fragments, in which downstream Met (Met54, Met108) were mutated, were obtained with the BioXP3200 DNA printer (sgidna.com/bxp3200) and subsequently cloned into the pB7FWG2 destination vector. The constructs were transformed in *A. thaliana* WT Col-0 plants via Agrobacterium-mediated floral dipping (Clough and Bent, 1998).

### Methyl viologen stress treatments

For the phenotypic analysis during methyl viologen (MV) stress, 2-week-old WT, *ANAC102.1* OE (OE1 and OE2), and *anac102* KO lines, grown together on a nylon mesh on ½ MS, were transferred to ½ MS medium supplemented with 2 μM MV (Sigma-Aldrich). The maximum photosystem II efficiency (Fv’/Fm’) was determined with a PAM-2000 chlorophyll fluorometer and the ImagingWinGigE software application (Walz; Effeltrich, Germany) on light-adapted plants. For RT-qPCR and RNA-sequencing (RNA-Seq) analyses on *ANAC102.1* OE and KO lines, the same setup was used, except for using a higher MV concentration (50 μM). For the RT-qPCR analysis of the *Met54-gANAC102-GFP OE* line and the *ANAC102* transcript isoforms, and for the western blot analysis of the ANAC102-GFP protein, 2-week-old seedlings grown on ½ MS medium were sprayed with 50 μM MV in water containing 0.1% Tween-20.

### RNA isolation and RT-qPCR analysis

Total RNA extraction from 100 mg of plant material was conducted using the ReliaPrep™ RNA Tissue Miniprep System (Promega) including DNase treatment. cDNA was synthesized from 1 μg of total RNA using the qScript cDNA SuperMix kit (Quanta BioSciences, Belgium), which includes a precise combination of Oligo(dT) and random primers. RT-qPCR analysis was performed using the SYBR Green I Master kit (Roche, Belgium) on a Roche LightCycler 480. The results were analyzed with the 2^-ΔΔCt^ method (Livak and Schmittgen, 2001). For the analysis of the *ANAC102* transcript models, two reference genes, *UBIQUITIN-CONJUGATING ENZYME 21* (*UBC21*) and *ACTIN7* (*ACT7*), were used for normalization. For the normalization of the *MDS* gene expression levels, *ACTIN-RELATED PROTEIN7* (*ARP7*) (*ANAC102.1* OE lines) or both *UBC21* and *ACTIN7* (*Met54-gANAC102-GFP* OE lines) were used. Stability of the expression of the reference genes was assessed under all experimental conditions (Supplementary Fig. S1). Results are based on four biological replicates for the transcript model analysis, two and three biological replicates for the *MDS* gene expression analyses in *ANAC102.1* OE and *Met54-gANAC102-GFP* OE lines, respectively. The RT-qPCR primers (Supplementary Table S1) were designed by the online Primer-BLAST tool (https://www.ncbi.nlm.nih.gov/tools/primer-blast/).

### Confocal imaging

For the subcellular localization analysis, the translational fluorescent reporter constructs were transformed into *Agrobacterium tumefaciens* GV3101 through electroporation. Subsequently, these *Agrobacterium* strains were infiltrated together with the RNA silencing inhibitor p19 in 4-week-old *N. benthamiana* epidermal cells and analyzed three days after infiltration (Li, 2011). Subcellular localization analysis in *Arabidopsis* lines stably transformed with the translational fluorescent reporter lines was performed on root tissue of 5-day-old seedlings. Imaging was performed on a Leica SP8 confocal microscope equipped with an HC PL APO CS2 40× water-corrected immersion objective. GFP was excited at 488 nm and captured between 515 and 545 nm. Chlorophyll autofluorescence was excited at 561 nm and detected at 560-630 nm.

### Protein extraction, immunoprecipitation and western blot analysis

One gram of plant tissue (whole seedlings of *Arabidopsis* and leaf tissue of *N. benthamiana*) was ground in liquid nitrogen and dissolved in protein extraction buffer (150 mM Tris-HCl pH 7.5, 150 mM NaCl, 1% (v/v) NP40, 10% (v/v) glycerol, 10 mM EDTA, 1 mM sodium molybdate, 1 mM PMSF, cOmplete™ protease inhibitor cocktail (Roche)). The homogenate was centrifuged for 30 min at 4°C to eliminate cell debris. The protein extract supernatant was collected and incubated with 40 μl GFP-Trap Magnetic Agarose beads (Chromotek) for 2 h at 4°C. The solution was removed and the beads were washed three times for 1 min with 700 μl washing buffer (20 mM Tris-HCl pH 7.5, 150 mM NaCl). Elution was performed by mixing the beads with 40 μl ultra-pure MQ H_2_O and 10 μl 5x Laemmli sample buffer (10% SDS, 25% 2-mercaptoehtanol, 50% glycerol,0.01%(w/v) bromophenol blue, 312.5 mM Tris HCl (pH 6.8)) and heating for 10 min at 95°C. Immunopurified protein eluates and total protein extracts were loaded and separated on a 4%-20% SDS-PAGE gel, transferred to PVDF membrane, blocked and blotted with anti-GFP-HRP (5000-fold dilution) (Miltenyi Biotec) using the Western Lightning kit (GE-Healthcare; http://www.gehealthcare.com/). Blots were imaged using BioRad Chemidoc system.

### Yeast one-hybrid (Y1H) screening

Yeast strain YM4271 and destination vector pMW#2 were obtained from Dr. M. Walhout (University of Massachusetts Medical School, Worcester, MA, USA). The REGIA collection was provided by Dr. Franziska Turck (Max Planck Institute for Plant Breeding Research, Köln, Germany). Design of the yeast reporter strains was done as described in detail (Deplancke *et al*., 2006). Primers used for cloning of the promoters are displayed in Supplementary Table S1. For the screening of the REGIA collection, the 1394 prey plasmids were individually transformed in the reporter yeast strains by means of a high-throughput transformation system in the 96-well format (Deplancke *et al*., 2006; Vermeirssen *et al*., 2007). Twenty μl of competent yeast cell suspension, 100 ng plasmid, and 100 μl Tris-EDTA (TE)/lithium acetate/polyethyleneglycol were combined per well. After heat shock (20 min at 30°C), plates were centrifuged (for 10 s) and supernatant was removed. Yeast cells were resuspended in 20 μl TE, of which 5 μl was spotted on selective (SD-His-Ura-Trp; Clontech) medium and on SD-His-Ura-Trp containing the appropriate concentration of 3-aminotriazole (3-AT) (Acros Organics) to minimize autoactivation. Growth on 3-AT was monitored during 3 to 10 days after transfer. Interactions were confirmed by retransforming the isolated prey plasmids in the yeast reporter strains and analyzing a 10- and 100-fold dilution on SD-His-Ura-Trp medium supplemented with 3-AT.

### RNA-sequencing sample preparation and data analysis

*ANAC102.1* OE, *anac102* KO and WT lines were sown together on a mesh placed on ½ MS medium and grown at a light intensity of 55-60 µmol/m^2^/s, with a light cycle of 16-h light/8-h dark. Subsequently, 2-week-old seedlings on the nylon mesh were transferred to ½ MS medium containing 50 µM MV or to normal ½ MS medium (mock). Shoot tissue of 5 to 6 seedlings was harvested for RNA extraction at 12 h after transfer. RNA concentration and purity were determined spectrophotometrically using a Nanodrop ND-1000 (Nanodrop Technologies) and RNA integrity was assessed using a Bioanalyzer 2100 (Agilent). Per sample, an amount of 1 µg of total RNA was used as input. Using the Illumina TruSeq® Stranded mRNA Sample Prep Kit (protocol version: Part # 1000000040498 v00 October 2017) poly-A-containing mRNA molecules were purified from the total RNA input using poly-T oligo-attached magnetic beads. In a reverse transcription reaction using random primers, RNA was converted into first strand cDNA and subsequently converted into double-stranded cDNA in a second strand cDNA synthesis reaction using DNA polymerase I and RNAse H. The cDNA fragments were extended with a single 'A' base to the 3' ends of the blunt-ended cDNA fragments, after which multiple indexing adapters were ligated introducing different barcodes for each sample. Finally, a PCR was carried out to enrich those DNA fragments that have adapter molecules on both ends and to amplify the amount of DNA in the library. Sequence-libraries of each sample were equimolarly pooled and sequenced on an Illumina NextSeq 500 (High Output Flowcell, 75 bp, Single Reads, v2.5) at the VIB Nucleomics Core (www.nucleomicscore.sites.vib.be). Data were processed using demultiplexing and quality controlled with FastQC. Alignment of reads was performed against the TAIR10 annotation using STAR (Dobin *et al*., 2013). On average 20 million reads per sample were mapped to the *Arabidopsis* genome. Counts were assigned to genes using featureCounts (Liao *et al*., 2014) and analysis of differentially expressed genes (DEGs) was performed with DeSEQ2, modeling for batch effects, without independent filtering and Cooks filter (Love *et al*., 2014). Transcripts were considered differentially expressed if pAdj < 0.05.

### Chromatin immunoprecipitation-sequencing (ChIP-Seq) data analysis

The ChIP-Seq peaks for *ANAC102* used in this study were adapted from Song *et al*. (2016). The peaks were annotated to the closest genes using the TAIR10 gene annotation, only considering protein-coding genes and discarding peaks that were >2000 bp away from the closest gene or that were overlapping with coding exons.

### Chlorophyll measurements

Total chlorophyll (Chl) was extracted from shoots of two-week-old *ANAC102.1* OE, *anac102* KO, and WT lines using 80% acetone and measured using a spectrophotometer at wavelengths of 645 nm and 663 nm according to Arnon (1949). The Chl index was measured based on reflectance at 710 and 770 nm (Gitelson *et al*., 2003) using a multispectral phenotyping platform. RGB (red green blue) images were processed via the “Data Analysis Software” program (Phenovation B.V., Wageningen, The Netherlands).

## Results

### ANAC102 *mainly expresses a nuclear protein isoform without a full-length N-terminal chloroplast-targeting peptide*

To evaluate and quantify various potential *ANAC102* transcript models, we analyzed different regions in the *ANAC102* genomic sequence via RT-qPCR. Four RT-qPCR primer pairs (qTSS1-1, qTSS1-2, qTSS2-1 and qTSS2-2; Supplementary Table S1) were designed downstream of the two TSSs (TSS1 and TSS2; Fig. 1A) previously identified by TSS-Seq (Ushijima *et al*., 2017; Nielsen *et al*., 2019), in addition to two upstream primer pairs encompassing the 5’ end of the *ANAC102.2* and *ANAC102.1* coding sequences in the TSS1 upstream region (qANAC102.2 CDS and qANAC102.1 CDS; Supplementary Table S1) and one targeting the 5’ untranslated region (q5’UTR; Fig. 1A; Supplementary Table S1). We analyzed shoot and root tissue from *Arabidopsis* WT seedlings grown under control conditions or treated with MV for 1, 3, 6 and 12 h. MV, which generates ROS mainly in the chloroplasts under light conditions, is generally considered as an inducer of chloroplast retrograde signaling and increases *ANAC102* transcript levels (Zimmermann *et al*., 2004; Ugalde *et al*., 2021). Under non-treated conditions (0h), the region downstream of the TSS1 (qTSS1-1) showed an approximately 15-fold (shoot tissue) and 61-fold (root tissue) higher expression compared to the region directly upstream of TSS1 (qANAC102.1 CDS) (Fig. 1B). This indicates that TSS1 is a predominant TSS, in agreement with a recent study (Cresta and D’Alessandro, 2023). Moreover, the 5’-extended transcripts (q5’UTR, qANAC102.2 CDS and qANAC102.1 CDS) in leaf tissue, overall showed a milder MV induction pattern compared to the earlier and stronger onset of the MV response of the shorter TSS1 and TSS2 transcripts (Fig. 1B). This is in agreement with the study by Cresta and D'Alessandro (2023), who investigated the responsiveness of different *ANAC102* isoforms to β-cc and photorespiratory stress, reporting no or a reduced induction of the larger, 5’-extended *ANAC102* isoform.

The transcript abundance of the region just downstream of TSS1 (qTSS1-1) under both control and stress conditions remained comparable to that of the more downstream qTSS1-2, implying the absence of additional TSSs between TSS1 and the second, downstream TSS2 (Ushijima *et al*., 2017). Analysis of the transcript abundance using primers targeting the TSS2 downstream region (qTSS2-2 and qTSS2-2) revealed higher (approximately 1.7-fold in shoot and 1.4-fold in root) expression levels compared to qTSS1-1 and qTSS1-2 (Fig. 1B), indicating that TSS2 might represent an additional TSS downstream of TSS1. However, the dynamics of stress induction were similar between TSS1 and TSS2 transcripts. In summary, TSS1 and TSS2 transcripts, both leading to protein isoforms that (likely) lack a functional cTP, are predominantly expressed, constituting 95% and 99% of the total transcript under control conditions in shoot and root tissue, respectively. Moreover, although TSS1 and TSS2 transcripts were the main isoforms under both control and stress conditions, a minor portion of the total transcript still encoded a larger, 5’-extended transcript, indicating that a chloroplastic ANAC102 proteoform might still exist.

To further unravel the 5’ extended transcript model(s), we analyzed the TSS1 upstream regions (q5’UTR, qANAC102.2 CDS and qANAC102.1 CDS). In shoot tissue, qANAC102.2 CDS and qANAC102.1 CDS showed similar expression levels, both under control and stress conditions, whereas the transcript levels corresponding to the 5’UTR region were considerably lower. In root tissue, qANAC102.1 CDS had ~2-3-fold higher transcript levels compared to both q5’UTR and qANAC102.2 CDS, which showed similar expression levels. These results indicate that different TSSs might exist in the *ANAC102* 5’ region and that these TSSs are differentially utilized between root and shoot tissue. However, the specifics of the protein models corresponding to the different transcript isoforms, including the translation initiation site (TIS) usage, remain unclear.

To evaluate the ANAC102 protein model, we used the full genomic *ANAC102* gene fragment [-1500 +1395] containing the 1.262-kb upstream regulatory region, the 5’UTR and all exons and introns, translationally fused to GFP (p*ANAC102*::g*ANAC102-GFP*). We analyzed the resulting protein isoform(s) in stably transformed p*ANAC102*::g*ANAC102-GFP Arabidopsis* lines, before and after a 1-, 3-, 6- and 12-h MV treatment. Protein immunoblot analysis of total protein extracts before and after enrichment by anti-GFP immunoprecipitation (IP) revealed only one protein isoform under both control and stress conditions (Fig. 1C). Comparison with different N-terminally truncated ANAC102 protein isoforms initiated at different in-frame start codons (Met11, Met54, Met108, Fig. 1A) after transient expression in *N. benthamiana* indicated translation likely occurs from Met54, the first in-frame AUG start codon downstream of TSS1. This protein isoform lacks the full cTP (Fig. 1A) and accordingly resulted in a solely nuclear localization pattern in roots of stably transformed p*ANAC102*::g*ANAC102*-GFP *Arabidopsis* plants and in leaves of *N. benthamiana* after transient expression of this construct (Supplementary Fig. S2). Although we could not detect any additional bands before and after enrichment by IP, under control nor MV stress conditions in which the abundance of *ANAC102* mRNA and protein was increased, we cannot exclude that a longer, N-terminal extended protein isoform is generated, albeit at levels we cannot detect with western blot performed on total protein extracts. Overexpression of the genomic fragment starting from Met11 (*Met11-gANAC102-GFP*) resulted in three protein bands (Fig. 1D) and in a dual chloroplast-nuclear localization in *N. benthamiana* leaves and a nuclear localization in *Arabidopsis* roots (Supplementary Fig. S2B). The largest protein isoform might correspond to the expected full-length Met11-gANAC102 protein (~63 kDa) whereas the slightly smaller proteoform might correspond to the chloroplast-imported protein with the cTP cleaved off. Although the two in-frame, downstream AUGs (Met54 and Met108) were mutated in the *Met11-gANAC102-GFP* construct, we cannot exclude that the observed short isoform is generated by alternative (non-AUG) translation initiation downstream of Met11, directly generating a cTP-depleted, nuclear isoform. Interestingly, the smallest, ~40 kDa proteoform, that was also detected in *Met108-gANAC102-GFP*, might be the result of translation from the TSS2 transcript. However, it was not obtained from the genomic p*ANAC102*::g*ANAC102* construct. Therefore, the protein isoform(s) that result from the TSS2 transcript and the question of whether they originate from the same open reading frame remain unresolved.

Our results suggest that Met54 is the predominant TIS resulting in a protein isoform containing the full NAC domain, but lacking the complete cTP. However, we cannot exclude that our method is not sensitive enough to detect N-terminally extended proteoform(s) resulting from translation from the 5’-extended mRNA that only represents a minor fraction of the total *ANAC102* transcript pool. Similarly, a previous ribosome profiling study identified AUG(Met54) as the predominant TIS in addition to an in-frame non-cognate (non-AUG) upstream TIS, AUU(Ile31) (Fig. 1 A, Supplementary Fig. S3) (Willems *et al*., 2022). This non-canonical TIS is also located downstream of TSS1, but it remains to be investigated whether this results in a proteoform with a functional cTP or alternatively produces a nuclear isoform. We hypothesize that the dominant protein isoform is the nuclear ANAC102 version translated from Met54 and therefore we will further focus on its function in the nucleus.

### ANAC102 *overexpression increases sensitivity to severe MV stress*

**Fig. 2:**
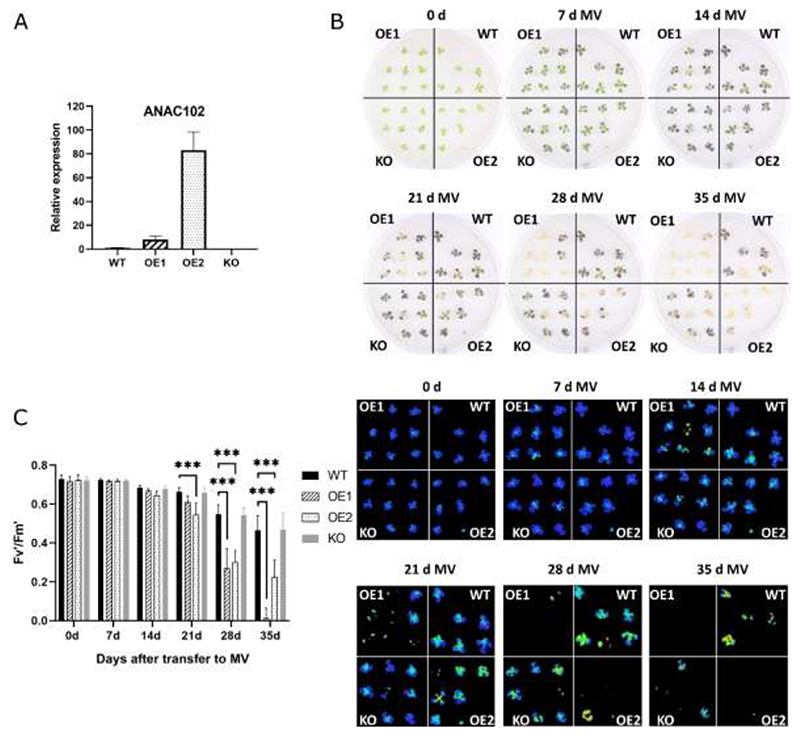
Phenotype of *ANAC102.1* OE and *anac102* KO plants under severe MV-induced oxidative stress. (A) Expression levels of *ANAC102* in *ANAC102.1* OE (OE1 and OE2) and *anac102* KO lines relative to WT. (B) Two-week-old wild-type (WT), independent transgenic *ANAC102* overexpression (OE1 and OE2) and *anac102* KO lines grown under control conditions were transferred to medium containing 2 μM methyl viologen (MV). Rosette growth was visually monitored from transfer to MV (0 d) until 35 days after transfer. (C) Quantification of the light-adapted efficiency of photosystem II (Fv’/Fm’) at different days after MV treatment and corresponding images. Error bars indicate the standard deviation (n = 15). Asterisks indicate significant differences to WT (*** *p*<0.001; two-way ANOVA with Tukey's multiple comparisons test).

Our previous findings demonstrated the involvement of ANAC102 in seedling growth during mild MV-induced oxidative stress conditions (De Clercq *et al*., 2021). To assess the performance of *ANAC102* gain- and loss-of-function lines under prolonged and more severe chloroplastic oxidative stress, we subjected *ANAC102.1* OE1 and OE2 [approximately 8-fold and 80-fold overexpression of the *ANAC102.1* (arabidopsis.org) CDS that includes the cTP (Inzé, 2012) (Fig. 2A)] and *anac102* knockout (KO; SALK_030702) plants to MV stress by transferring 2-week-old seedlings to medium supplemented with 2 µM MV. Rosette growth and photosystem II efficiency (Fv’/Fm’) were monitored at seven-day intervals. No significant differences were observed between WT and the *anac102 KO* line (Fig. 2B and C). Results revealed that starting from day 21 post treatment, both *ANAC102.1* OE1 and OE2 began to exhibit signs of distress, ultimately leading to their demise at 5 weeks post treatment, in contrast to prolonged survival of the WT and *anac102* KO plants (Fig. 2B). Additionally, Fv’/Fm’ values of *ANAC102.1* OE lines, and especially their younger leaves, showed a significant decline from 21 d after treatment compared to both the WT and *anac102* KO (Fig. 2C).

Since the *ANAC102.1* OE lines overexpress both the chloroplastic and nuclear ANAC102 proteins (Supplementary Fig. S2), we evaluated whether the MV sensitivity phenotype is due to increased activity of ANAC102 in the nucleus. We repeated the MV stress assays on p*35S*::*Met54-gANAC102-GFP* lines that solely overexpress the cTP-depleted, nuclear isoform to a tenfold (Supplementary Fig. S4). Similar to the *ANAC102.1* OE lines, *Met54-gANAC102-GFP* OE lines displayed earlier and more severe stress symptoms compared to WT plants, evidenced by accelerated bleaching of the plants and a decreased Fv’/Fm’ from 2 weeks after transfer to MV (Supplemental Fig. S4). In summary, overexpression of *ANAC102* increases sensitivity to severe MV-induced oxidative stress and this phenotype is mainly caused by ANAC102 activity in the nucleus.

### ANAC102 is a repressor of MV-induced retrograde gene expression

**Fig. 3:**
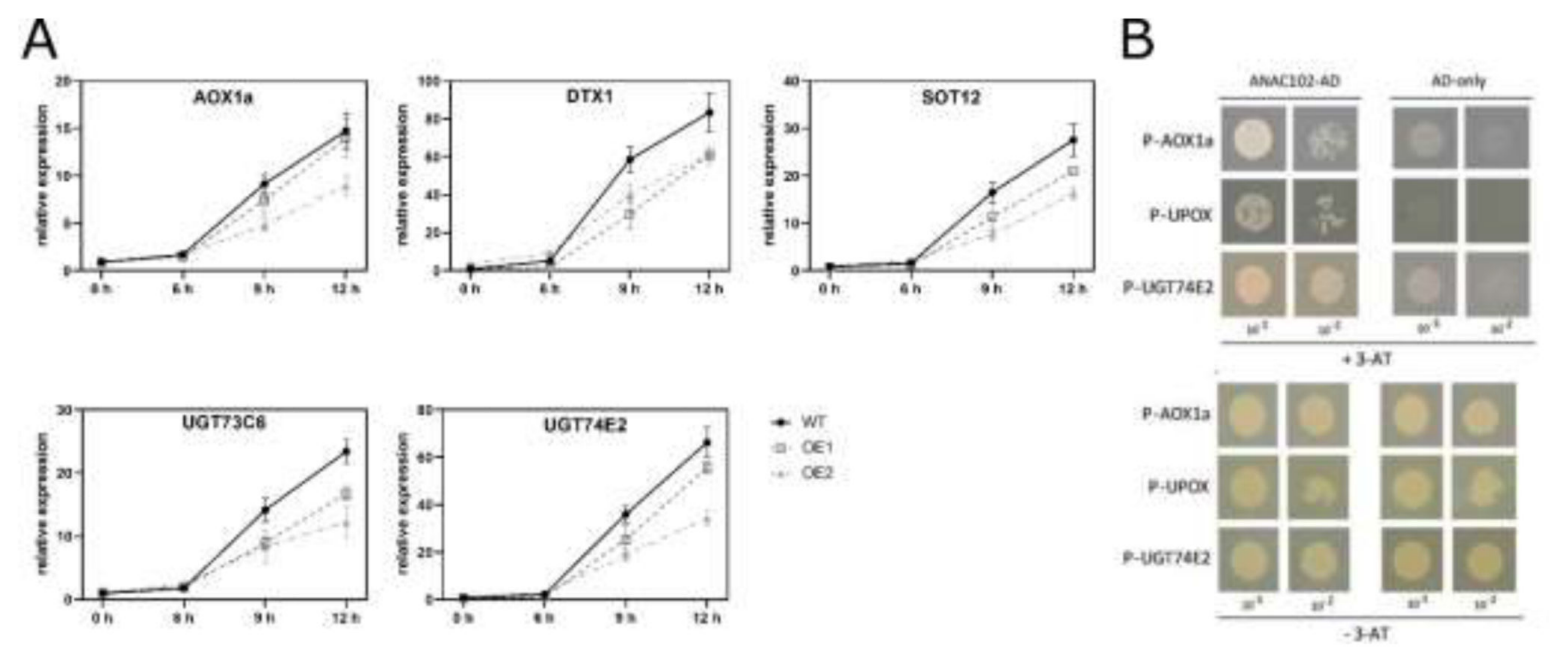
ANAC102 negatively regulates chloroplast retrograde regulation of nuclear gene expression. (A) RT-qPCR analysis of retrograde target gene expression in wild-type (WT) and two independent *ANAC102.1* OE lines (OE1, ~8-fold and OE2, ~80-fold) during an MV treatment time course. Error bars indicate the standard deviation (n = 2 biological replicates). Similar data were obtained in at least two other biological repeat experiments. (B) Yeast one-hybrid analysis illustrating the binding of ANAC102 to retrograde target gene promoters. Yeast reporter strains, each containing the *HIS3* reporter under the control of the *AOX1a, UPOX* and *UGT74E2* promoters and transformed with either ANAC102 fused to the GAL4 activation domain (ANAC102-AD) or the AD only (AD), were grown in the presence or absence of minimal 3-AT concentrations (5 – 20 mM) to suppress autoactivation.

Subsequently, we investigated whether MV-responsive genes in the nucleus are modulated in *ANAC102* gain-of-function lines. Two-week-old WT and *ANAC102.1* OE1 and OE2 were transferred to medium supplemented with 50 μM MV, and shoots were harvested before (0 h) and at various time points (6, 9 and 12 h) after MV treatment for RT-qPCR analysis. We assessed the expression of *mitochondrial dysfunction stimulon* (*MDS*) genes that were previously defined to be common targets of mitochondrial and MV-induced chloroplast retrograde signaling and that are regulated through ANAC013, a regulator that positively impacts plant MV stress tolerance (De Clercq *et al*., 2013; Van Aken *et al*., 2016). As expected, *MDS* genes (*AOX1a, DTX1, SOT12, UGT74E2* and *UGT73C6*) were induced by MV in both WT and *ANAC102.1* OE1 and OE2 lines and induction was prominent from 9 h after treatment (Fig. 3A). However, a comparative analysis between WT and *ANAC102.1* OE lines indicated a partial repression of the MV-induced expression of the *MDS* genes in the strong OE2 line and to a lesser extent in the weaker OE1 line. This indicates that *ANAC102.1* OE partially represses MV responses in the nucleus and that the repression effect occurred in a dose-dependent manner.

To further reveal whether perturbed retrograde responsive gene expression is due to ANAC102 activity in the nucleus, we analyzed *MDS* gene expression in p*35S*::*Met54-gANAC102-GFP* lines. Similar to the results obtained with *ANAC102.1* OE lines, *MDS* gene induction by MV was partially repressed in *Met54-gANAC102-GFP* OE when compared to the WT (Supplementary Fig. S5).

We identified ANAC102 in a Y1H screen for TFs binding to retrograde target gene promoters, including the *AOX1a, UGT74E2* and *UPOX* promoters, using the REGIA library of Arabidopsis TFs (Paz-Ares and The REGIA Consortium, 2002). We specifically reassessed and confirmed these interactions in targeted Y1H assays, demonstrating that ANAC102 can indeed directly bind to the promoters of *AOX1a, UGT74E2* and *UPOX* (Fig. 3B). Moreover, analysis of available ChIP-Seq data (Song *et al*., 2016) (Materials and methods) indicated binding of ANAC102 to *AOX1a* and *UGT74E2* promoters in both plants grown with or without abscisic acid treatment, but not to the *DTX1, SOT12, UGT73C6* and *UPOX* promoters.

In conclusion, our study demonstrates that ANAC102 acts as a repressor of MV-induced retrograde gene expression, potentially by directly binding to the target promoters in the nucleus.

### ANAC102 negatively and positively regulates genome-wide transcriptional responses to MV stress

**Fig. 4:**
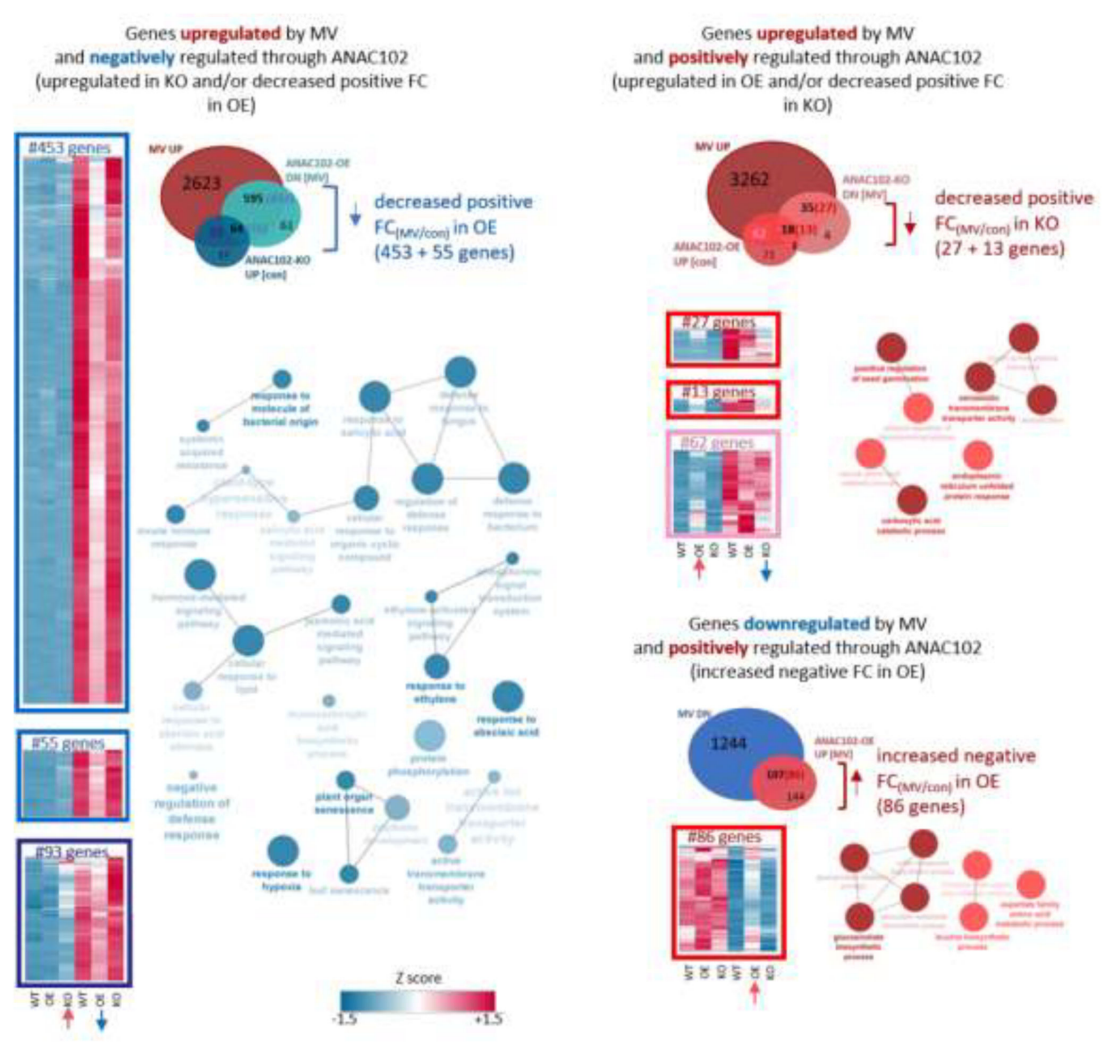
ANAC102 negatively and positively regulates MV responses. MV responsive genes that are differentially regulated through ANAC102 were identified and classified in three clusters based on their up- or down-regulation by MV and their positive or negative regulation through ANAC102. Venn diagrams display the number of genes that are regulated by MV in WT (FC > 1.5 fold between MV and control in WT, FDR < 0.05) and/or that are differentially regulated by ANAC102 (FC >1.3 fold between *ANAC102.1* OE2 or *anac102* KO and WT, FDR < 0.05) under control (con) or MV stress conditions. Numbers between brackets in the Venn diagram intersections indicate genes that in addition have a differential (> 1.25) MV-responsive fold change (FC_[MV/con]_) between OE2 or KO and WT. Their expression profiles are displayed in heatmaps (Z score of normalized count data; blue, low expression levels; red, high expression levels) and their functional enrichment (based on biological process Gene Ontology (GO) terms) was analyzed with ClueGO (Bindea *et al*., 2009). Enrichment significance of the GO term is reflected by the node color (darker color indicating higher significance) and the size of colored nodes indicates the number of genes mapped to each GO term.

To assess whether and how ANAC102 affects genome-wide transcriptional responses to MV, an RNA-Seq analysis was performed on *ANAC102.1* OE2, *anac102* KO and WT plants that were mock-treated or treated with MV. Since MV-induced *MDS* gene expression was significantly induced at 12 h of MV treatment (Fig. 3A), we analyzed the genome-wide MV responses 12 h after treatment. The transcript levels of 3375 and 1351 genes were induced and repressed by MV in WT plants, respectively (FC > 1.5; FDR < 0.05). A significant overlap was observed between MV-induced and -downregulated genes in *ANAC102.1* OE2 vs WT under MV stress conditions (659/720 *ANAC102.1* OE2 downregulated genes, hypergeometric *P* value = 0) and/or genes upregulated in *anac102* KO under control conditions (157/174 *anac102KO* upregulated genes, hypergeometric *P* value = 7.35E-135), indicating that ANAC102 negatively regulates (part of) the MV-induced transcriptome (Fig. 4). Among the 3375 genes induced by MV in WT, 18% were negatively regulated by ANAC102, i.e. having both a decreased positive fold change_(MV/control)_ (> 1.25 fold difference in FC) and lower MV-induced expression levels in *ANAC102.1* OE2 vs WT (>1.3 fold, FDR < 0.05) and/or having higher basal transcript levels in *anac102* KO vs WT(>1.3 fold, FDR < 0.05). These MV-induced genes that were negatively regulated through ANAC102 include mitochondria/chloroplast retrograde target genes, such as *AOX1a, SOT12, UGT74E2, UPOX, AT5G43450, AT2G32020, CYP81D8, ABCB4* and *AT2G04050*, as well as the well-established retrograde regulator *ANAC013* (De Clercq *et al*., 2013). Gene Ontology (GO) enrichment analysis indicated enrichment of various functions, including plant defense response to biotic stress, hormone (salicylic acid, ethylene, jasmonic acid, abscisic acid) signaling, response to hypoxia stress and glucosinolate metabolism. Among these DEGs, there were 73 TFs including members of the WRKY and ERF families with functions in biotic stress responses [(WRKY48; Xing *et al*., 2008) and (ERF1A; Cheng *et al*., 2013)], as well as TFs involved in abscisic acid signaling (MYB30; Zheng *et al*., 2012), hypoxia (ERF71; Hess *et al*., 2011) and glucosinolate catabolism (BGLU28; Zhang *et al*., 2020).

In addition to MV-induced genes negatively regulated by ANAC102, there was a small gene set (3%) that was upregulated by MV and positively regulated through ANAC102. These genes had elevated basal expression levels in *ANAC102.1* OE2 (> 1.3 FC, FDR < 0.05) and/or showed a decreased MV induction (> 1.25 fold difference in FC) as well as lower MV-induced gene expression levels in *anac102* KO vs WT (> 1.3 FC, FDR < 0.05). Accordingly, a significant overlap was observed between MV-upregulated genes and genes downregulated in *anac102* KO under MV stress (53/58 of the *anac102* KO downregulated genes, hypergeometric *P* value = 8.08E-47) and between MV-upregulated genes and genes upregulated in *ANAC102.1* OE2 under control conditions (80/152 of the *ANAC102.1* OE2 upregulated genes, hypergeometric *P* value = 6.18E-39) (Fig. 4). These MV-induced genes positively regulated through ANAC102 were enriched for functions in xenobiotic detoxification, transmembrane transport activity and unfolded protein response (UPR) and included, among other TFs, bZIP60 with a well-established function in the regulation of UPR genes (Ye *et al*., 2011; Moreno *et al*., 2012).

On the other hand, ANAC102 affected the MV-downregulated transcriptome with 86/1351 (6%) MV-repressed genes being positively regulated by ANAC102, i.e. having an increased negative FC_[MV/control]_ in *ANAC102.1* OE2 (> 1.25 difference in FC) and increased expression levels in *ANAC102.1* OE2 (> 1.3 FC. FDR < 0.05) vs WT under MV stress. Accordingly, there was a significant overlap between the MV-downregulated transcriptome and *ANAC102.1* OE2 upregulated genes under MV stress (107/251 of *ANAC102.1* OE2 upregulated genes, hypergeometric P value = 3.75E-80) (Fig. 4). These MV-downregulated genes positively regulated through ANAC102 were enriched for functions in glucosinolate biosynthesis and response to insects, and included, among other TFs, MYB29, a regulator of glucosinolate biosynthesis genes that has also been implicated as a negative regulator of retrograde signaling through regulating the complex interplay between hormone and ROS signaling (Gigolashvili *et al*., 2008; Zhang *et al*., 2017).

A previous study associated an altered Chl content with increased chloroplastic ANAC102 levels in OE lines of *ANAC102.1* CDS fused to a nuclear export sequence (Xin *et al*., 2021). Since an altered Chl content could affect the photosynthesis rate and ROS levels induced by MV, we measured whether our (dual chloroplast-nuclear targeted) *ANAC102.1* CDS OE1 and OE2, (nuclear targeted) *Met54-gANAC102-GFP* OE and *anac102* KO lines also had altered Chl concentrations. Although the Chl content determined after Chl extraction was not significantly changed in any of the *ANAC102* mutant lines (Supplementary Fig. S6A), the Chl index estimation (Gitelson *et al*., 2003) was significantly decreased in *ANAC102.1* OE2 and significantly increased in *anac102* KO lines relative to the WT (Supplementary Fig. S6B). These results indicate that we cannot exclude that part of the dampened nuclear transcriptional responses to MV might be because of the *ANAC102.1* OE2 and *anac102* KO seedlings being, respectively, more desensitized or sensitized to MV.

To summarize, while ANAC102 positively regulates a small subset of the MV-upregulated transcriptome, a substantial portion of the MV-responsive transcriptome was dysregulated in *ANAC102.1* OE lines. Specifically, this dysregulation manifests as diminished induction of MV-upregulated genes and attenuated repression of MV-downregulated genes.

### ANAC102 regulates transcriptional MV responses directly and indirectly through downstream TF networks

**Fig. 5:**
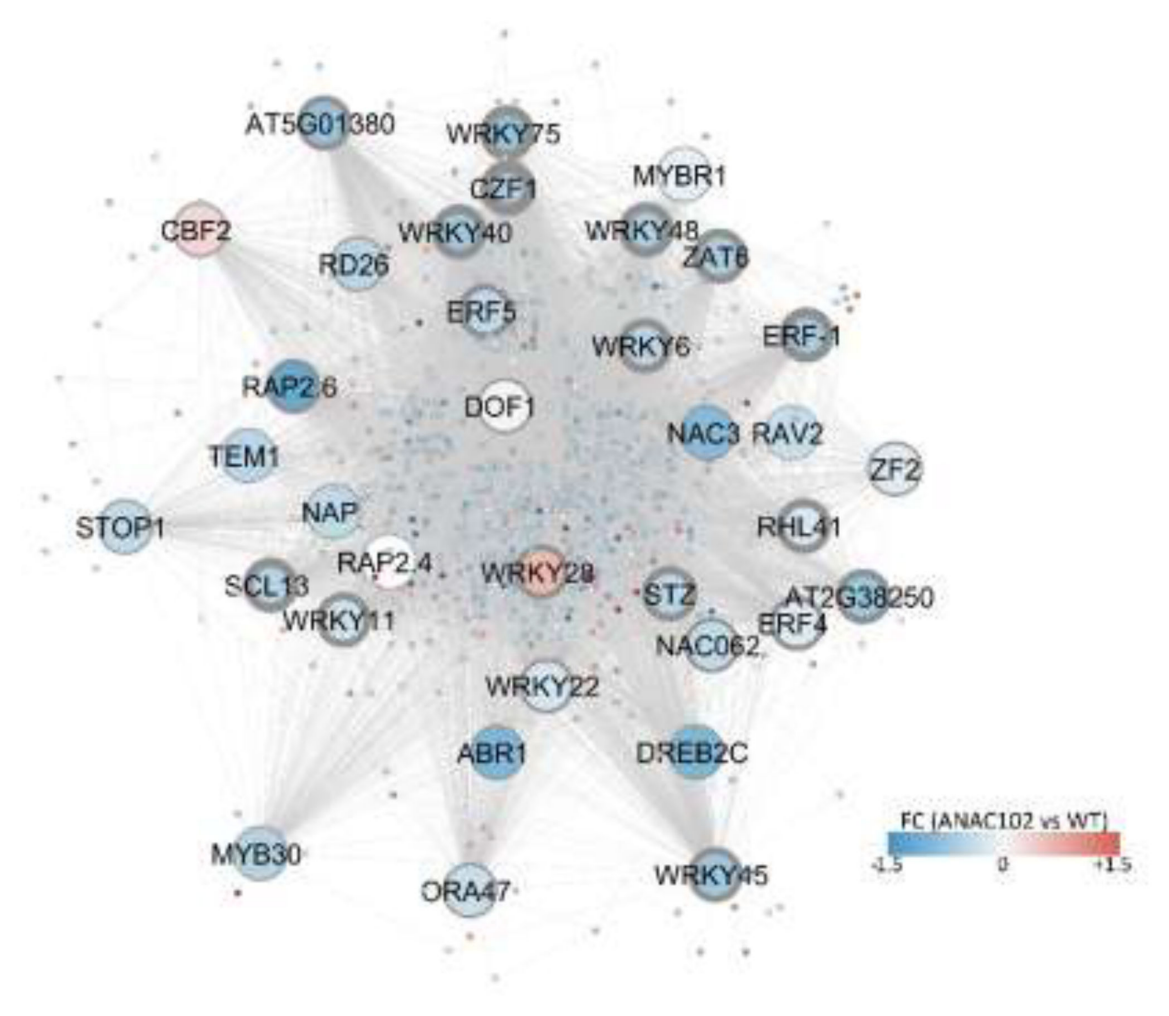
Transcriptional regulatory networks mediating MV responses downstream of ANAC102. To construct transcriptional regulatory networks modulated by ANAC102 in response to MV stress, we identified MV-responsive TFs exhibiting differential expression in *ANAC102.1* OE2 and/or *anac102* KO (RNA-Seq) that were also classified as direct ANAC102 targets based on ChIP-Seq analysis (Song *et al*., 2016) and that were additionally predicted by iGRN (De Clercq *et al*., 2021) as regulators of ANAC102 DEGs. The TF (large nodes) – target gene (small nodes) interactions were displayed with Cytoscape 3.8.2 (Shannon *et al*., 2003). Thickness of the TF node border lines indicate enrichment statistics (q-value based on hypergeometric distribution combined with Benjamini-Hochberg correction (De Clercq *et al*., 2021)) of TF association with the target genes. Node color indicates the fold change in *ANAC102.1* OE2 compared to WT under MV stress or the negative FC of *anac102* KO compared to WT under MV stress, with blue indicating negative and red indicating positive regulation by ANAC102.

To investigate the underlying mechanisms governing the differential regulation of MV responses in *ANAC102* gain- or loss-of-function lines, we sought to determine whether ANAC102 exerts direct transcriptional control or if its influence is mediated indirectly through downstream TF networks. We conducted an analysis by intersecting DEGs with the direct target genes identified through ANAC102 ChIP-Seq analysis (Song *et al*., 2016) (peak annotation procedure detailed in Materials and methods). Among the MV-induced genes negatively regulated by ANAC102, 50% were also identified as direct target genes in the ChIP-Seq dataset. Similarly, 52% of the genes positively regulated by ANAC102 during MV induction and 24% of the genes positively regulated by ANAC102 during MV repression, overlapped with ANAC102 ChIP-Seq target genes. These observations suggest that a substantial portion of the MV-responsive transcriptome under ANAC102 regulation may be directly modulated through ANAC102’s binding to the promoters. As the overlap between ANAC102-regulated DEGs and ChIP-Seq data revealed 39 TFs, we explored whether these TFs could act as intermediates in regulating the ANAC102-regulated transcriptome under MV stress. Using TF2Network that predicts TFs for a set of target genes based on motif enrichment, TF–target gene co-expression and experimental protein–DNA binding data (Kulkarni *et al*., 2018), we predicted 24 of these TFs to regulate a subset of the ANAC102-regulated transcriptome during MV stress (Supplementary Fig. S7). Similarly, employing a more advanced gene regulatory network approach, integrating DNA motif, open chromatin, TF binding and co-expression information using a machine-learning [referred to as integrated gene regulatory network (iGRN), De Clercq *et al*. (2021)], we predicted 34 of these 39 ANAC102-regulated TFs as regulators of ANAC102 DEGs, with 10 TFs (CZF1, WRKY75, WRKY48, ERF-1, SCL13, WRKY40, ZAT6, RHL41, WRKY6, STZ) among the top 20 most significantly enriched TFs and CZF1, WRKY75, WRKY48 being ranked first, third and fourth, respectively (Fig. 5). Interestingly, 17 TFs were predicted by both TF2Network and iGRN (WRKY75, WRKY48, ERF-1, WRKY40, WRKY6, WRKY11, ERF5, WRKY45, WRKY28, ERF4, ANAC062, WRKY22, ORA47, DOF1, CBF2, ABR1, MYB30), indicating that in addition to direct transcriptional regulation of target genes, ANAC102 plays a role in orchestrating downstream TF regulatory networks, primarily by exerting negative regulation on these networks (Fig. 5).

Among the putative intermediate TFs were several TFs with functions in plant abiotic and biotic stress responses and more specifically with functions related to ANAC102. For example, SALT TOLERANCE ZINC FINGER (STZ), analogous to ANAC102, has a positive regulatory role in photooxidative stress responses (Rossel *et al*., 2007). In addition, WRKY40 and WRKY45 confer tolerance to hypoxia through retrograde signaling and are involved in age-triggered leaf senescence, and both these functions were enriched among the MV-upregulated genes negatively regulated by ANAC102 (Fig. 5) (Chen *et al*., 2017; Meng *et al*., 2020). SCARECROW-LIKE 13 (SCL13) is a positive regulator of phytochrome-dependent red light signaling (Torres-Galea *et al*., 2006). Interestingly, *ANAC102* transcription initiation is differentially regulated by phytochrome-dependent red light through alternative promoter selection and its closest homolog, *ATAF2*, has been implicated in photomorphogenesis (Ushijima *et al*., 2017). Moreover, several of the identified TFs have functions in response to microbial pathogens [for example, (WRKY11, Jiang *et al*. (2016) and (WRKY48, Xing *et al*. (2008)]. Although the role of ANAC102 in response to biotic stress factors has to our knowledge not been studied, many of its DEGs are involved in biotic stress responses and glucosinolate biosynthesis (Fig. 4). However, its close relatives, ATAF1 and ATAF2, have well-established functions in defense responses and ATAF2 has also been implicated in the response to wounding (Delessert *et al*., 2005; Wang *et al*., 2009).

## Discussion

### ANAC102 *mainly expresses a nuclear proteoform without the complete N-terminal cTP*

In this study, our primary objective was to clarify the subcellular localization of ANAC102 that was previously implicated in the orchestration of plant responses to various stresses (Christianson *et al*., 2009; D’Alessandro *et al*., 2018; Han *et al*., 2023). Previous studies that investigated the annotated *ANAC102* coding sequences (*ANAC102.1* and *ANAC102.2*) driven by a constitutive promoter underlined its localization and function in chloroplasts (Inzé *et al*., 2012; Xin *et al*., 2021). Our study, however, used the full genomic *ANAC102* sequence including its 5’ regulatory and 5’UTR region and revealed under both control and stress conditions only one protein isoform that lacked a complete N-terminal cTP and solely localized to the nucleus (Fig. 1 and Supplementary Fig. S2). This proteoform likely arises from the first TIS (Met54) downstream of a TSS identified by TSS-Seq (Ushijima *et al*., 2017; Nielsen *et al*., 2019) and previously identified by RiboSeq profiling (Willems *et al*., 2022). However, as our and other recent studies (Cresta and D'Alessandro, 2023; Han *et al*., 2023) additionally detected a larger, 5’-extended mRNA isoform, albeit at very low concentrations, we cannot exclude that a proteoform with a full, functional cTP also exists. When we enforced transcription of *ANAC102* from a more upstream start codon by a constitutive promoter (*p35S::Met11-gANAC102-GFP*), we observed both a nuclear and chloroplast localization, as (Xin *et al*., 2021) did. However, it remains unclear how the dual localization is achieved and whether the nuclear isoform is obtained after relocalization of the chloroplast-imported protein or is a result of dual targeting of the full-length protein from the cytosol as observed for SWIB-4 (Melonek *et al*., 2012). Although the two in-frame, downstream AUGs (Met54 and Met108) were mutated in the *Met11-gANAC102-GFP* construct, we cannot exclude that a cTP-depleted, nuclear isoform is directly generated as a result of alternative (non-AUG) translation initiation downstream of Met11. Nevertheless, a constitutive promoter driving expression of a specific transcript model might not be representative of the actual physiological situation. To conclude, our study supports that *ANAC102* primarily expresses a nuclear isoform and therefore mainly exerts a function in the nucleus. This is in agreement with a recent study showing a function of ANAC102 as a direct transcriptional activator of a nuclear Cd-responsive gene (Han *et al*., 2023).

### ANAC102 *overexpression increases sensitivity to MV-induced chloroplast oxidative stress*

ANAC102 was previously identified as a regulator of low-oxygen, cadmium and β-cc-mediated photooxidative stress responses, exerting a positive effect on tolerance to these stresses (Christianson *et al*., 2009; D’Alessandro *et al*., 2018; Han *et al*., 2023). Accordingly, *ANAC102* transcript levels were induced under these stress conditions. However, induction by Cd and β-cc was not uniform throughout the plant, but restricted to the root and young leaves, respectively. In our previous study, we reported an altered growth phenotype by *ANAC102* overexpression after germination and growth on mild (nM range) MV stress, with contradicting phenotypes depending on the overexpression levels (De Clercq *et al*., 2021). Here, we show that both weak and strong *ANAC102* OE lines are more sensitive to long-term, more severe (μM range) MV stress, resulting in an accelerated cell death phenotype (Fig. 2). Moreover, our findings reveal that *ANAC102* overexpression partially suppresses the upregulation of MV-induced genes, including a set of well-established retrograde target genes, potentially through direct binding to their promoters (Fig. 3). These retrograde target genes, previously referred to as *MDS* genes, include members of cytochrome P450 monooxygenases, glutathione S-transferases, UDP-glucosyl transferases and transmembrane transporters that have putative functions in detoxification and transport of toxic and/or reactive molecules. Moreover, the *MDS* genes are direct targets of ANAC013 and ANAC017 that are known to positively influence plant MV tolerance (De Clercq *et al*., 2013; Van Aken *et al*., 2016). In contrast, our transcriptome analysis as well as the study by D’Alessandro *et al*. (2018) show that ANAC102 also positively regulates a subset of genes involved in detoxification responses. Together, our and previous studies underscore the complex role of ANAC102 in fine-tuning the plant's responses to environmental challenges, particularly to stresses generating reactive molecules such as hypoxia, heavy metals, excess light and chemicals inducing the generation of ROS in the chloroplasts. However, our study introduces a new perspective to the understanding of ANAC102's function, that might not only act as an activator of stress responses, but also as a repressor depending on the promoter context or the severity and/or type of stress condition. A previous study indicated that *ANAC102* is mainly induced in young leaves, and that the β-cc-mediated detoxification responses regulated through SCL14, and potentially ANAC102, contribute to the protection and the higher resistance of young leaves against high-light stress (D’Alessandro *et al*., 2018). In contrast, in our MV stress assays, we observed an increased sensitivity of the young leaves compared to older leaves, resulting in a faster decline in photosynthetic activity and accelerated cell death (Fig. 2). Moreover, the MV sensitivity of young leaves is more pronounced in *ANAC102* OE lines compared to the WT, indicating that under severe MV stress, ANAC102 might negatively modulate detoxification responses in young tissues. Altogether, our study underscores the complex role of ANAC102 in positively and negatively regulating detoxification responses depending on the type and severity of the stress and the specific tissue and/or developmental stage of the plant.

### ANAC102 regulates MV-induced chloroplast retrograde responses in the nucleus

Previous studies have demonstrated that changes in ANAC102 levels impact nuclear expression, particularly of those genes involved in low oxygen and metal stress responses, in detoxification during photooxidative stress and in brassinosteroid metabolism during seedling photomorphogenesis (Christianson *et al*., 2009; D’Alessandro *et al*., 2018; Peng and Neff, 2020; Han *et al*., 2023). Moreover, the promoters of genes differentially expressed by *ANAC102* overexpression have been found to overrepresent a NAC consensus binding site, suggesting that ANAC102 directly regulates nuclear gene expression (Christianson *et al*., 2009). In addition, ANAC102 was shown to activate a nuclear gene involved in Cd stress tolerance by directly binding to its promoter (Han *et al*., 2023). In this study, we further confirmed its nuclear function by demonstrating that ANAC102 binds to the promoters of the *MDS* retrograde target genes in the Y1H system and by comparative analysis of the ANAC102-regulated MV-responsive transcriptome and ANAC102 nuclear target genes identified by ChIP-Seq analysis. However, we cannot exclude that part of the altered MV-responsive transcriptome by *ANAC102* overexpression or knockout is indirect and a result of physiological changes from altered *ANAC102* expression; for example, altered Chl levels in *ANAC102* OE and KO (Xin *et al*., 2021) (Supplementary Fig. S6) could affect light absorption, thereby (de)sensitizing plants to MV.

Our transcriptome analysis further unraveled a dual role for ANAC102 in modulating genome-wide transcriptional responses to MV stress by mediating both negative and positive regulation, thereby influencing the MV-controlled up- and downregulation of a diverse set of genes (Fig. 4). This dual regulatory function suggests a sophisticated mechanism by which ANAC102 acts as both a transcriptional activator and repressor and this might depend on the promoter context, growth/stress conditions and/or the developmental stage of the plants. Similarly, its close relative, ATAF2, has been shown to act as an activator or repressor, activating or repressing *PATHOGENESIS-RELATED GENE* gene expression, depending on the specific growth conditions and developmental stage of the plants (Delessert *et al*., 2005; Wang *et al*., 2009). Although we observed a substantial overlap between ANAC102-negatively regulated genes and ANAC102 target genes identified by ChIP-Seq, a transcriptional repressor function remains to be proven. ANAC102 has previously been shown to suppress the expression of two brassinosteroid catabolic genes, but this was not through direct promoter binding, but likely through protein–protein interactions with ATAF2 and CCA1 that act as direct repressors of these genes (Peng *et al*., 2015; Peng and Neff, 2020). Thus part of the complex regulatory mechanisms of ANAC102 might be by homo- and heterodimer formation among/with the ATAFs and other TFs such as CCA1. Interestingly, several of the ANAC102-regulated DEGs identified during MV responses overlapped with ATAF2 target genes identified during control and wounding-stress conditions (Delessert *et al*., 2005). As we previously observed contradicting phenotypes between weak and strong *ANAC102* OE lines during mild MV stress (De Clercq *et al*., 2021), ANAC102 might act as an activator or repressor in a dose-dependent manner. Similarly, the *Drosophila* zinc finger TF KRUPPEL can have opposite regulatory effects on the expression of a single gene in a concentration-dependent manner, converting from an activator to repressor at higher concentrations through homodimer formation (Sauer and Jäckle, 1993).

Together, our ChIP-Seq analysis and reverse engineering of TF networks indicates that ANAC102 influences the MV responses via both direct and indirect regulatory interactions through downstream TFs, which (Fig. 5) are mostly negatively regulated by ANAC102. Several of these TFs could contribute to reported ANAC102 functions, including photooxidative and hypoxia stress responses (Rossel *et al*., 2007; Meng *et al*., 2020). This complex regulatory network provides insights into the putative role of ANAC102 as a master regulator in modulating plant responses to various environmental challenges. To summarize, our study suggests a sophisticated mechanism by which ANAC102 fine-tunes the plant's transcriptional landscape during various stresses through a dual activator and repressor function and through the regulation of downstream TF regulatory networks.

## Supplementary Material

Supplementary Materials

## Data Availability

The data supporting the findings of this study are available from the corresponding author upon request.

## Highlight

We revealed the multifaceted role of ANAC102 as both a positive and negative regulator of chloroplast oxidative stress responses through complex gene regulatory networks in the nucleus.

## Abbreviations

(β-cc): β-cyclocitral
(Cd): cadmium
(p35S): Cauliflower Mosaic Virus 35S promoter
(Chl): chlorophyll
(ChIP-Seq): chromatin immunoprecipitation-sequencing
(cTP): chloroplast-targeting peptide
(DEGs): differentially expressed genes
(GO): Gene Ontology
(IP): immunoprecipitation
(KO): knockout
(OE): overexpressing
(MV): methyl viologen
(MDS): mitochondrial dysfunction stimulon
(MS): Murashige and Skoog
(OE): overexpression
(RT-qPCR): quantitative real-time PCR
(ROS): reactive oxygen species
(RNA-Seq): RNA-sequencing
(TF): transcription factor
(TSS): transcription start site
(TIS): translation initiation site
(TE): Tris-EDTA
(WT): wild type
(Y1H): yeast one-hybrid
