# Supplementary material for: ANAC102 predominantly expresses a nuclear protein and acts as a negative regulator of methyl viologen-induced retrograde signaling
Source: J Exp Bot. Author manuscript; Available in PMC 2025 Feb 12. (PMC7616362; doi:10.1093/jxb/erae235)
Supplement: Supplementary Materials [file EMS196787-supplement-Supplementary_Materials.docx]

**Supplementary Table S1.** List of primers used in this study.

| **Primer name** | **Prime sequence 5'->3'** | **Application** |
| --- | --- | --- |
| attB1_ANAC102[-1500  +1395]_FW | GGGGACAAGTTTGTACAAAAAAGCAGGCTCCACCAAGACTTGGACCCAACACAGCC | Gateway cloning |
| attB2_ANAC102[-1500  +1395]_RV | GGGGACCACTTTGTACAAGAAAGCTGGGTCCCCTTGAGGAGCAAAATTCCAA | Gateway cloning |
| q5'UTR_qFW | GTCCAAGCCACGTGTAAACC | ANAC102 5'UTR RT-qPCR |
| q5'UTR_qRV | TTCTTTCGGACACGTGTGAT | ANAC102 5'UTR RT-qPCR |
| qANAC102.1 CDS_qFW | ACTTTGCTCTCTTCTCCTCGAT | AANAC102.1 CDS RT-qPCR |
| qANAC102.1 CDS_qRV | AAGTACCTTGTTGTTTTCTTGTGG | ANAC102.1 CDS RT-qPCR |
| qANAC102.2 CDS_qFW | TCCCCTTCGTTTATAAAAGCTCC | ANAC102.2 CDS RT-qPCR |
| qANAC102.2 CDS_qRV | TCGAATCGGAGGATCTTTGTGG | ANAC102.2 CDS RT-qPCR |
| qTSS1-1_qFW | ACCACAAAGATCCTCCGATTC | ANAC102 TSS1-1 RT-qPCR |
| qTSS1-1_qRV | CGCCTTCATTTTTGGAAAAGTACC | ANAC102 TSS1-1 RT-qPCR |
| qTSS1-2_qFW | CGTTCCGGTTATCGCAGAGA | ANAC102 TSS1-2 RT-qPCR |
| qTSS1-2_qRV | TTTCTCACCGTACAACGCCA | ANAC102 TSS1-2 RT-qPCR |
| qTSS2-1_qFW | ATGTCGATCGATCTGCTTCTACC | ANAC102 TSS2-1 RT-qPCR |
| qTSS2-1_qRV | ATCCGACACAAAACCCAATCA | ANAC102 TSS2-1 RT-qPCR |
| qTSS2-2_qFW | TGACGCCTTTGTTCCTCAGT | ANAC102 TSS2-2 RT-qPCR |
| qTSS2-2_RV | GCTCGAACGGGTCCTGAAA | ANAC102 TSS2-2 RT-qPCR |
| UBC21_qFW | TTGTGCCATTGAATTGAACCC | RT-qPCR reference genes |
| UBC21_qRV | CTGCGACTCAGGGAATCTTCTA | RT-qPCR reference genes |
| ACT7_qFW | GGAAACATCGTTCTCAGTGGT | RT-qPCR reference genes |
| ACT7_qRV | CTTGATCTTCATGCTGCTAGGT | RT-qPCR reference genes |
| ARP7_qFW | ACTCTTCCTGATGGACAGGTG | RT-qPCR reference genes |
| ARP7_qRV | CTCAACGATTCCATGCTCCT | RT-qPCR reference genes |
| at2g36790-qFW | TGCCGAGGTTAAAGAGGTCA | UGT73C6 RT-qPCR |
| at2g36790-qRV | TCCACCAACACTCCTATCTTCTC | UGT73C6 RT-qPCR |
| at2g03760-qFW | GGTCACCAATCCACACCTTC | SOT12 RT-qPCR |
| at2g03760-qRV | CGAAATCTGGGGACTCGTAG | SOT12 RT-qPCR |
| at1g05680-qFW | GAATCGTCCTCATACCCGAAT | UGT74E2 RT-qPCR |
| at1g05680-qRV | GCTTTGGACCCATTTCAACA | UGT74E2 RT-qPCR |
| at2g04040-qFW | CATCAGCTGCAATGATTTGTC | DTX1 RT-qPCR |
| at2g04040-qRV | GAACAGAGGTCTCGAGTTTCG | DTX1 RT-qPCR |
| at2g21640-qFW | AAAAGAAAGAAGAAGCGAAGAAGA | UPOX RT-qPCR |
| at2g21640-qRV | CTTGGTATCCACCACGGTTT | UPOX RT-qPCR |
| SALK_030702_LP | CTTTTCCAAAAATGAAGGCG | T-DNA genotyping |
| SALK_030702_RP | TGAGCATCTTGAGTCCGACG | T-DNA genotyping |
| SALK_LB | ATTTTGCCGATTTCGGAAC | T-DNA genotyping |
| AttB4_P-UPOX_FW | ATAGAAAAGTTGACAACATTGATCATACGAGATCAAAAAGG | Y1H promoter cloning |
| AttB1r_P-UPOX_RV | TGTACAAACTTGACGCTGAAAACAGAAAGAAATCTCATGAA | Y1H promoter cloning |
| AttB4_P-UGT74E2_FW | GGGGACAACTTTGTATAGAAAAGTTGGATTTCACCCATGATATACTG | Y1H promoter cloning |
| AttB1r_P-UGT74E2_RV | GGGGACTGCTTTTTTGTACAAACTTGTTTCTCCTTCTTTTTAATCTTGT | Y1H promoter cloning |
| AttB4_P-AOX1a_FW | ATAGAAAAGTTGATCTGAAGAGCTTCTAGC | Y1H promoter cloning |
| AttB1r_P-AOX1a _RV | TGTACAAACTTGTGTTTCAAATCGGAAAAAGTG | Y1H promoter cloning |

**
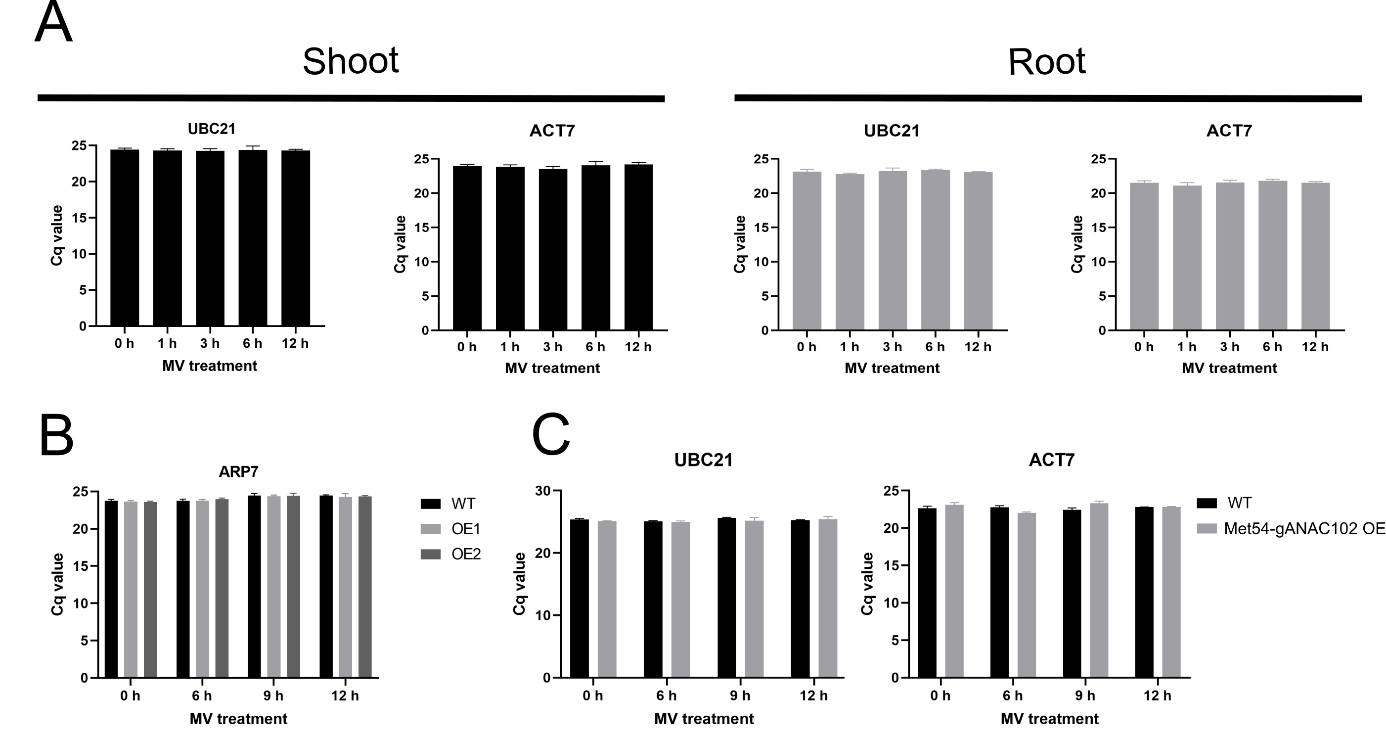
**

**Supplementary Figure S1.** **Quantification cycle (Cq) values of the reference genes in the RT-qPCR samples.**(A) Cq values of *UBC21* and *ACT7*used to correct for transcript abundance inthe MV treatment time course experiment in WT shoot and root (Figure 1A). Error bars indicate the standard deviation (n = 4 biological replicates). (B) Cq values of *ARP7*used to correct for transcript abundance in the MV treatment time course in WT and *ANAC102.1* OE (OE1 and OE2) (Figure 3A). Error bars indicate the standard deviation (n = 2 biological replicates). (C) Cq values of *UBC21* and *ACT7* used to correct for transcript abundance in the MV treatment time course experiment in WT and *Met54-gANAC102-GFP*OE shoot. Error bars indicate the standard deviation (n = 3 biological replicates).


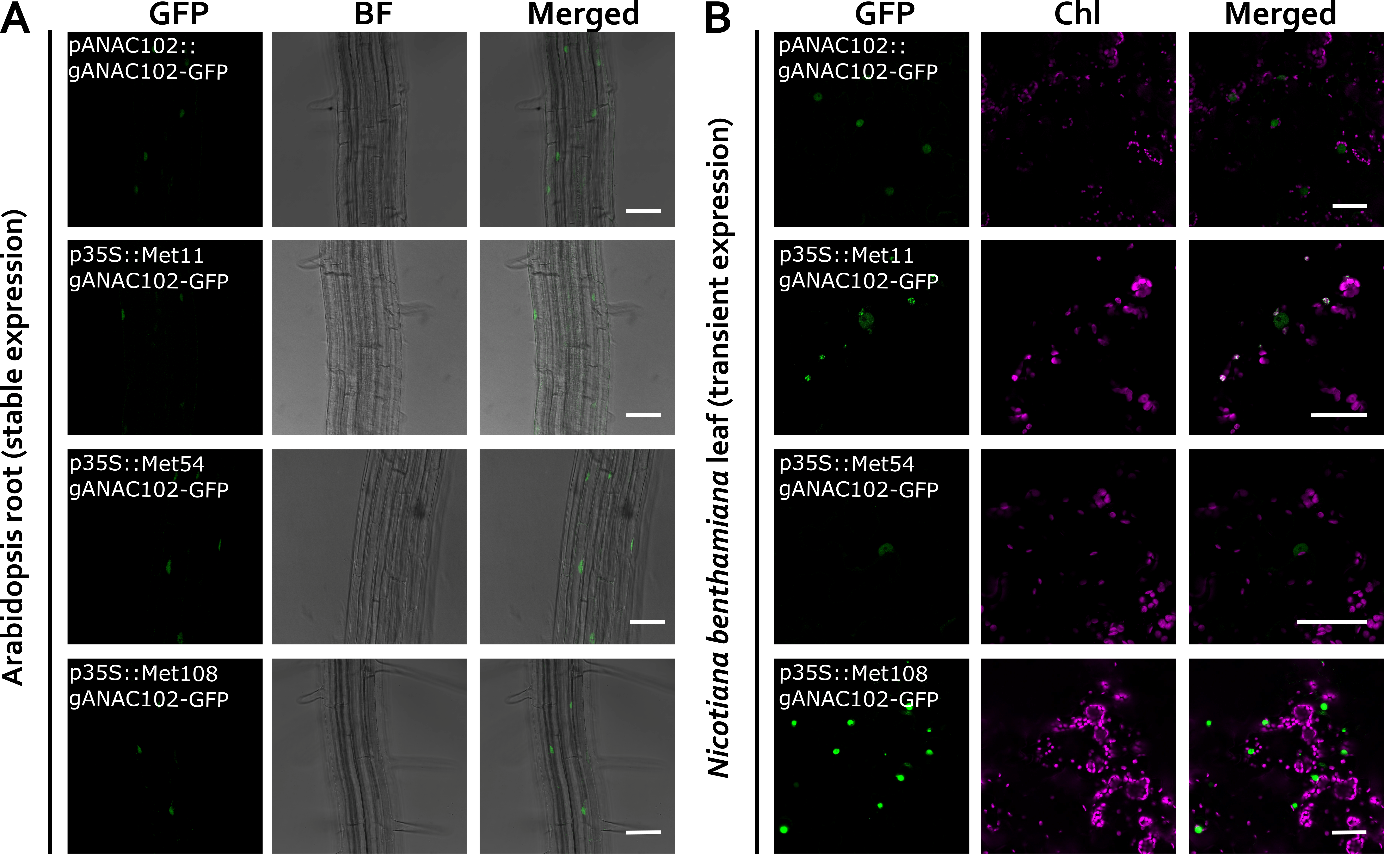


**Supplementary Figure S2.** **Subcellular localization of gANAC102-GFP and comparison with N-terminal truncated gANAC102 proteoforms.**

(A) Subcellular localization of gANAC102-GFP in roots of 5-day-oldstable transgenic *Arabidopsis* lines expressing *pANAC102::gANAC102-GFP*, *p35S::Met11-gANAC102-GFP*, *p35S::Met54-gANAC102-GFP* and *p35S::Met108-gANAC102-GFP*, scale bars = 50 µm. BF, birght field; Chl, chorophyll autofluorescence. (B) Subcellular localization of the same constructs after transient expression in *N. benthamiana*leaf epidermal cells,scale bars = 50 µm.Chl, chlorophyll autofluorescence.


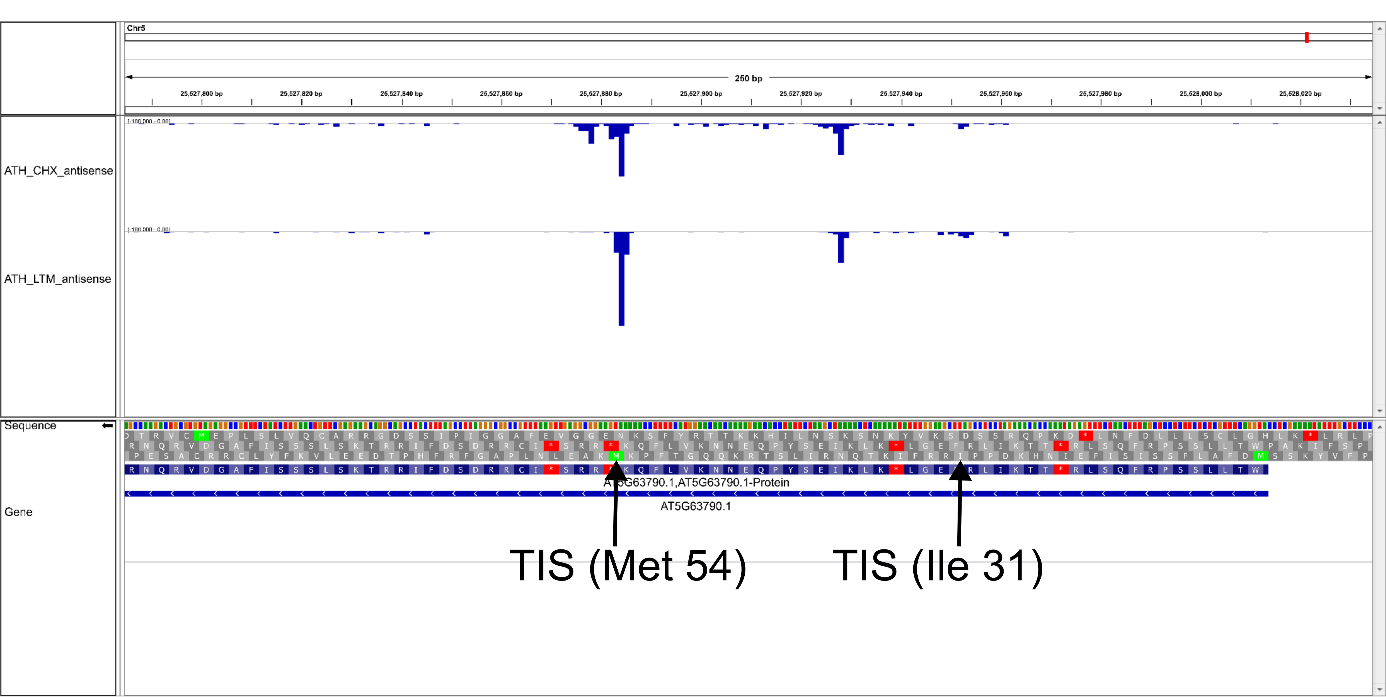


**Supplementary Figure S3.** **Ribosome profiling results of *ANAC102* translation initiation sites according to Willems *et al.* (2022).**Data files from GSE88790 were visualized in the Integrative Genome Viewer. Identified, in-frame translation initiation sites (TIS) are indicated with arrows.


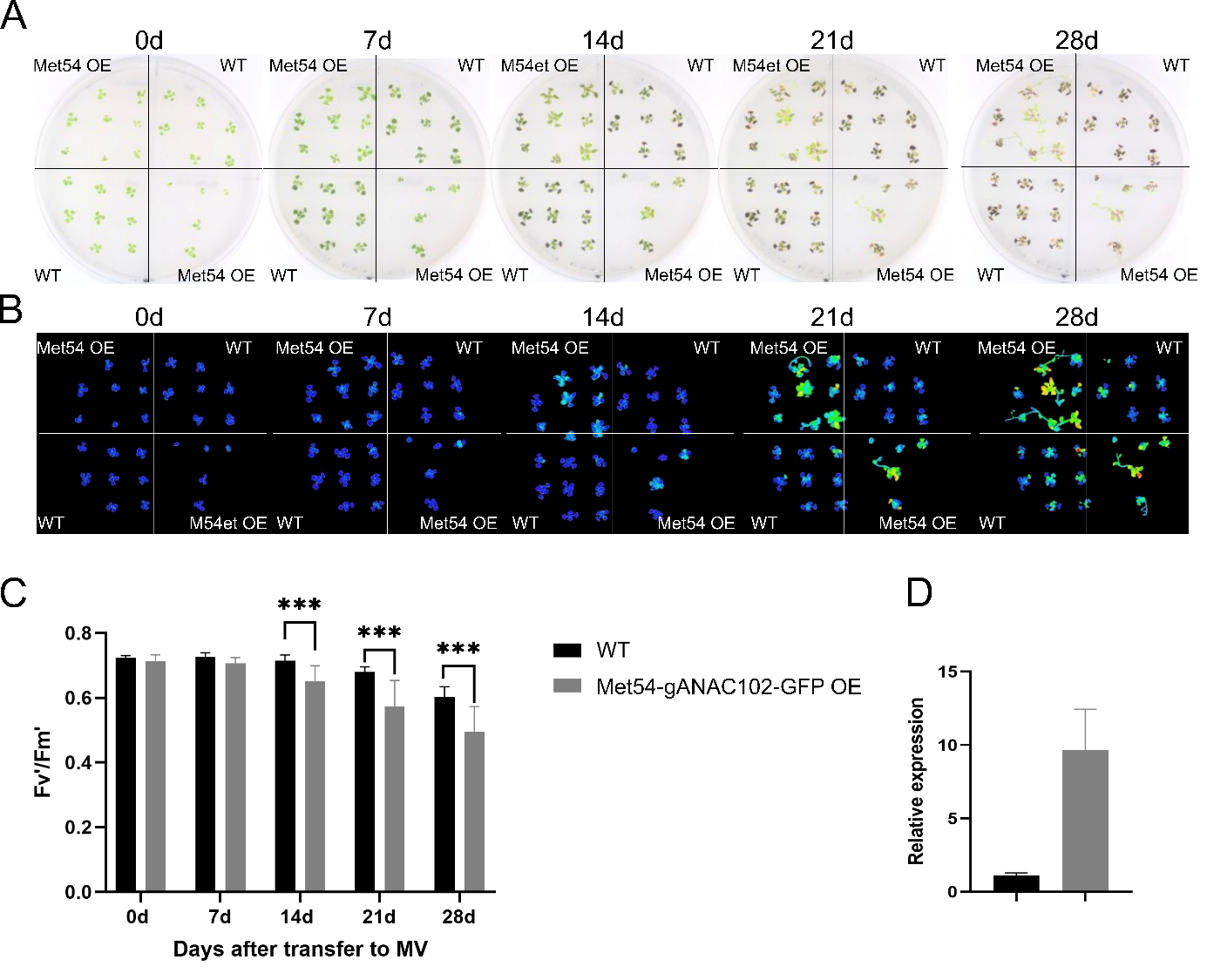


**Supplementary Figure S4.****Phenotype of*Met54-gANAC102-GFP* OE plants under MV-induced oxidative stress.**(A) Two-week-old wild-type (WT) and*Met54-gANAC102-GFP* overexpression (OE)lines grown under control conditions were transferred to medium containing 2 μM methyl viologen (MV). Rosette growth was visually monitored from transfer to MV (0 d) until 28 days after transfer. (B) Images of light-adapted quantum efficiency of photosystem II (Fv’/Fm’). (C) Quantification of Fv’/Fm’. Error bars indicate the standard deviation(n = 15). Asterisks indicate significant differences to the WT (*** *p*<0.001; two-way ANOVA with Tukey's multiple comparisons test).(D) Expression levels of *ANAC102* in *Met54-*g*ANAC102* OE lines relative to WT.


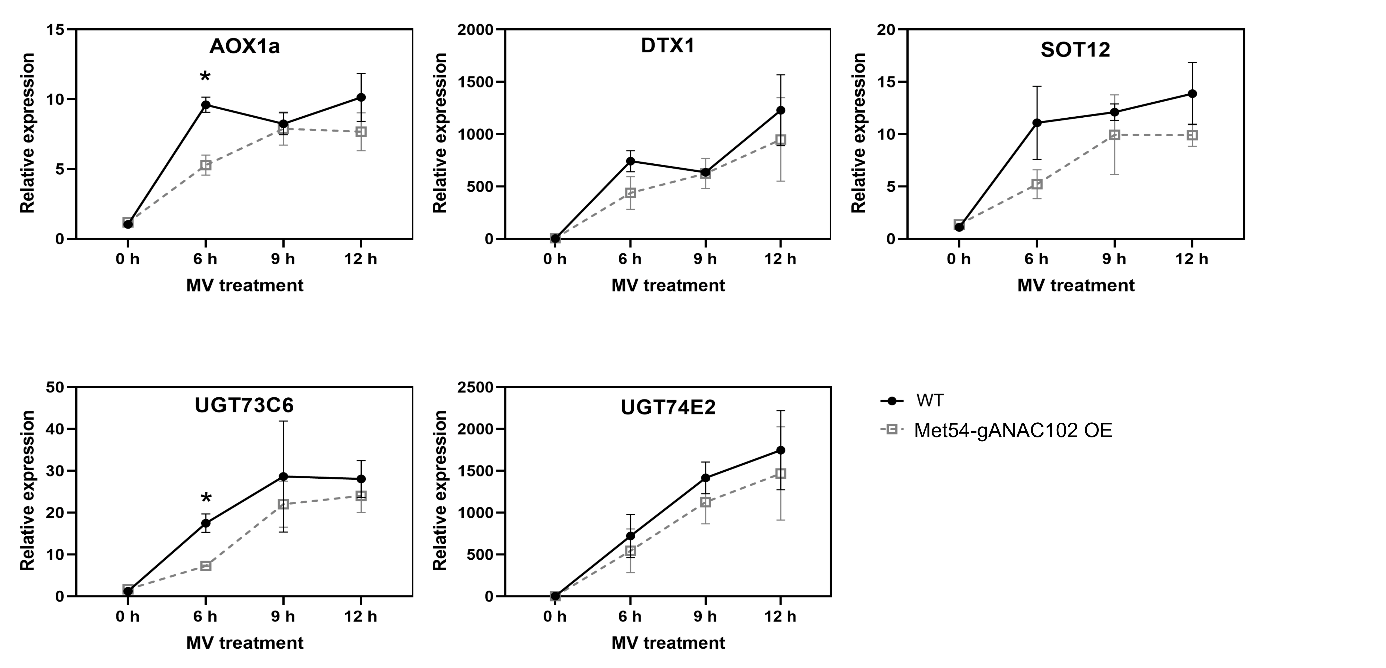


**Supplementary Figure S5.*****MDS* gene expression in the *Met54-gANAC102* OE line.**

Two-week-old WT and*Met54-gANAC102-GFP*OElineswere sprayed with 50 μMMV. *MDS* gene expression was measured at 6, 9 and 12 h after MV-induced oxidative stress. Error bars indicate the standard deviation(n = 3 biological replicates), **p*<0.05, two-way ANOVA with Tukey's multiple comparisons test.


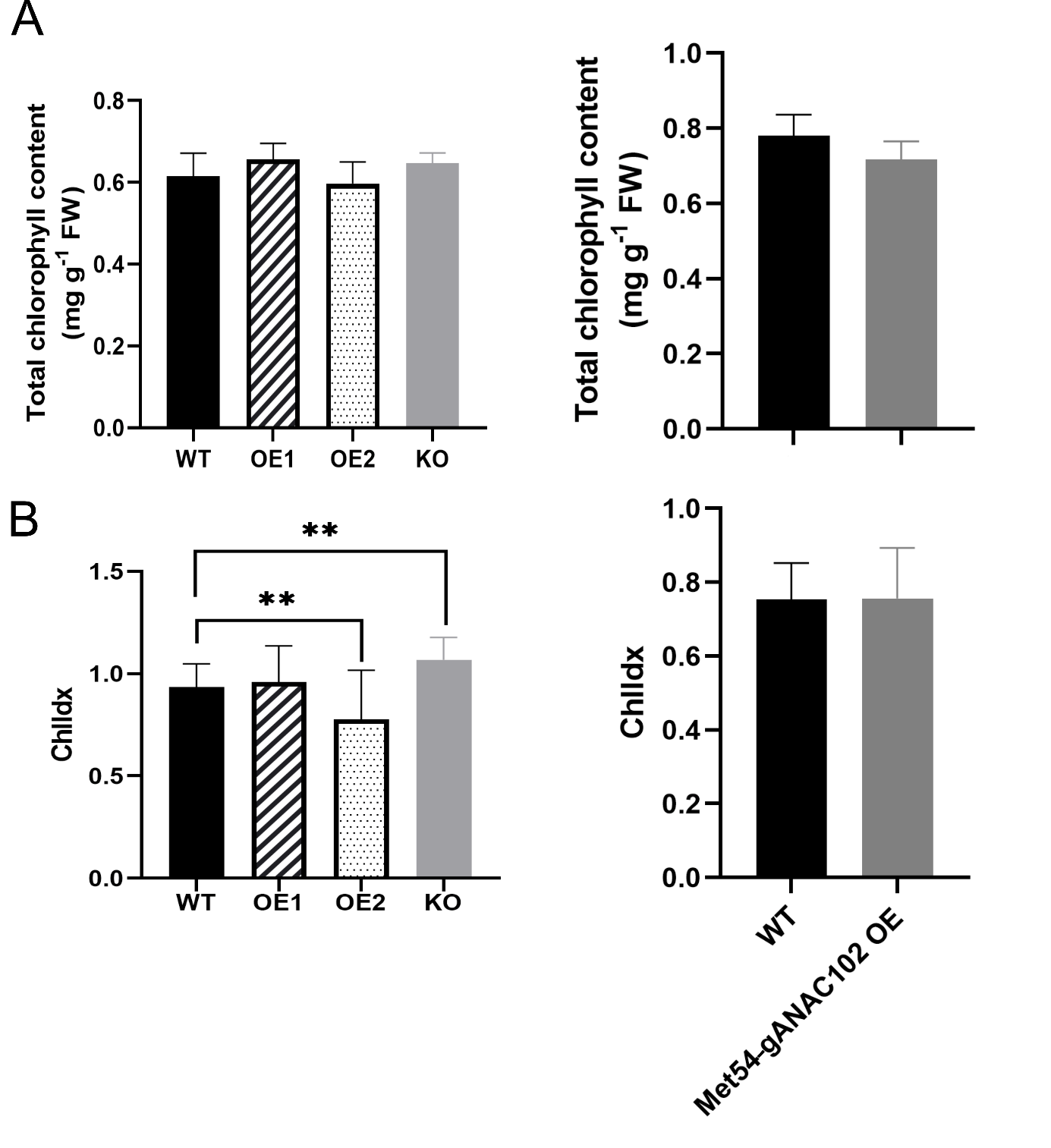


**Supplementary Figure S6.****Total chlorophyll content and chlorophyll index of WT and *ANAC102* OE and KO lines.**(A)Total chlorophyll content (mg/g fresh weight) in WT, *ANAC102.1* OE (OE1 and OE2), *anac102* KO, and *Met54-gANAC102* OE lines. The bars represent averages ± the standard deviation(n = 3). (B)Chlorophyll index (Chlldx) measurements in WT, *ANAC102.1* OE (OE1 and OE2), *anac102* KO, and *Met54-gANAC102* OE lines. The bars represent averages ± the standard deviation(n = 20 plants).***p*<0.01, Wilcoxon signed-rank test.


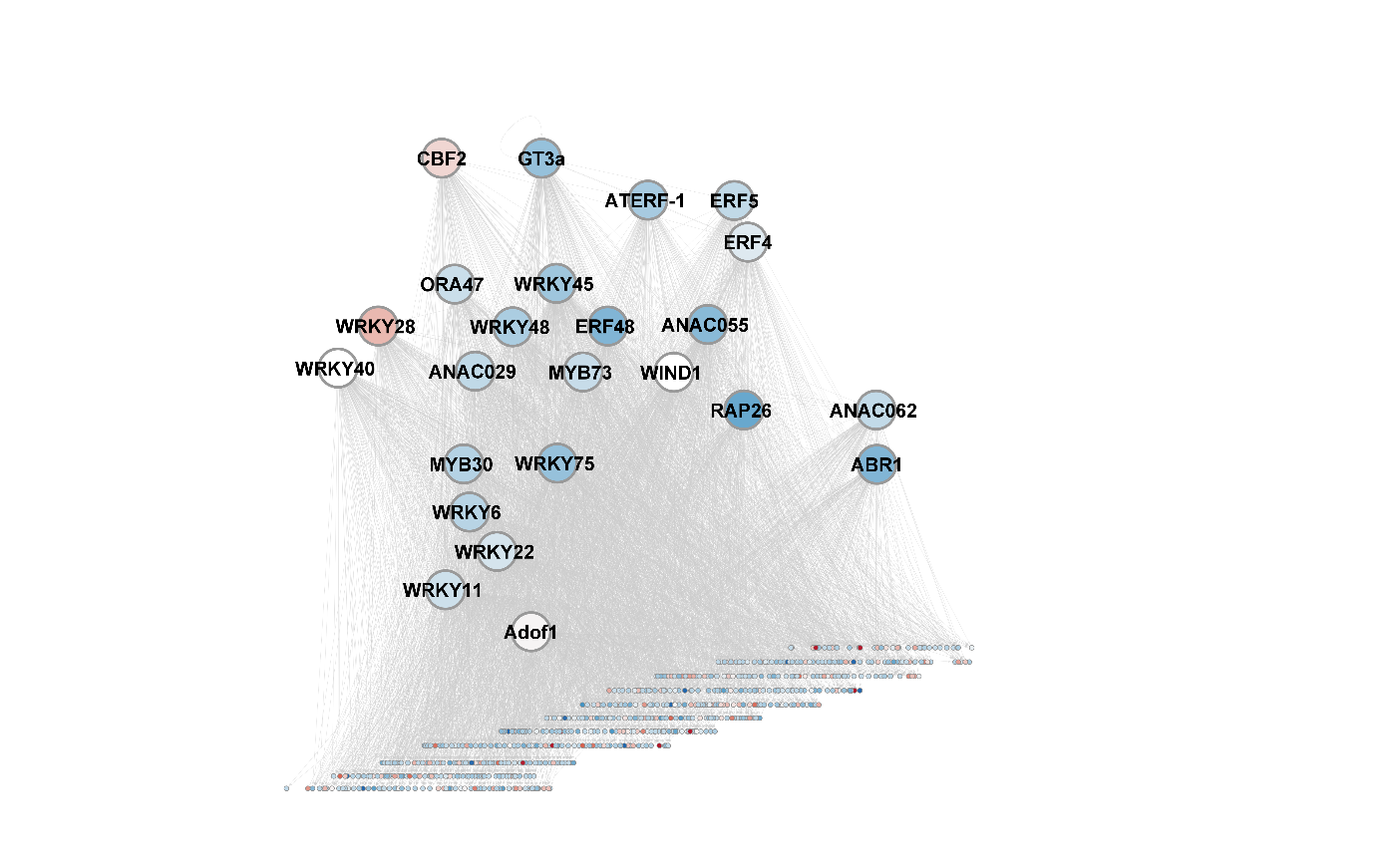


**Supplementary Fig. S7.** **Construction of the transcriptional regulatory network mediating MV responses downstream of ANAC102 using TF2Network.** To construct transcriptional regulatory networks modulated by ANAC102 in response to MV stress, we identified MV-responsive TFs exhibiting differential expression in *ANAC102* OE2 and/or *anac102* KOlines (RNA-Seq) that were also classified as direct ANAC102 targets based on ChIP-Seq analysis (Song *et al.*, 2016) and that were additionally predicted by TF2Network (Kulkarni *et al.*, 2018) as regulators of ANAC102-regulated DEGs. The TF (large nodes)–target gene (small nodes) interactions were displayed with Cytoscape 3.8.2 (Shannon *et al.*, 2003). Node color indicates the fold change in *ANAC102* OE2 compared to WT under MV stress or the negative FC of *anac102* KO compared to WT under MV stress, with blue indicating negative and red indicating positive regulation by ANAC102.

**Supplementary Table S1.** List of primers used in this study.

| **Primer name** | **Prime sequence 5'->3'** | **Application** |
| --- | --- | --- |
| attB1_ANAC102[-1500  +1395]_FW | GGGGACAAGTTTGTACAAAAAAGCAGGCTCCACCAAGACTTGGACCCAACACAGCC | Gateway cloning |
| attB2_ANAC102[-1500  +1395]_RV | GGGGACCACTTTGTACAAGAAAGCTGGGTCCCCTTGAGGAGCAAAATTCCAA | Gateway cloning |
| q5'UTR_qFW | GTCCAAGCCACGTGTAAACC | ANAC102 5'UTR RT-qPCR |
| q5'UTR_qRV | TTCTTTCGGACACGTGTGAT | ANAC102 5'UTR RT-qPCR |
| qANAC102.1 CDS_qFW | ACTTTGCTCTCTTCTCCTCGAT | ANAC102.1 CDS RT-qPCR |
| qANAC102.1 CDS_qRV | AAGTACCTTGTTGTTTTCTTGTGG | ANAC102.1 CDS RT-qPCR |
| qANAC102.2 CDS_qFW | TCCCCTTCGTTTATAAAAGCTCC | ANAC102.2 CDS RT-qPCR |
| qANAC102.2 CDS_qRV | TCGAATCGGAGGATCTTTGTGG | ANAC102.2 CDS RT-qPCR |
| qTSS1-1_qFW | ACCACAAAGATCCTCCGATTC | ANAC102 TSS1-1 RT-qPCR |
| qTSS1-1_qRV | CGCCTTCATTTTTGGAAAAGTACC | ANAC102 TSS1-1 RT-qPCR |
| qTSS1-2_qFW | CGTTCCGGTTATCGCAGAGA | ANAC102 TSS1-2 RT-qPCR |
| qTSS1-2_qRV | TTTCTCACCGTACAACGCCA | ANAC102 TSS1-2 RT-qPCR |
| qTSS2-1_qFW | ATGTCGATCGATCTGCTTCTACC | ANAC102 TSS2-1 RT-qPCR |
| qTSS2-1_qRV | ATCCGACACAAAACCCAATCA | ANAC102 TSS2-1 RT-qPCR |
| qTSS2-2_qFW | TGACGCCTTTGTTCCTCAGT | ANAC102 TSS2-2 RT-qPCR |
| qTSS2-2_RV | GCTCGAACGGGTCCTGAAA | ANAC102 TSS2-2 RT-qPCR |
| UBC21_qFW | TTGTGCCATTGAATTGAACCC | RT-qPCR reference genes |
| UBC21_qRV | CTGCGACTCAGGGAATCTTCTA | RT-qPCR reference genes |
| ACT7_qFW | GGAAACATCGTTCTCAGTGGT | RT-qPCR reference genes |
| ACT7_qRV | CTTGATCTTCATGCTGCTAGGT | RT-qPCR reference genes |
| ARP7_qFW | ACTCTTCCTGATGGACAGGTG | RT-qPCR reference genes |
| ARP7_qRV | CTCAACGATTCCATGCTCCT | RT-qPCR reference genes |
| at2g36790-qFW | TGCCGAGGTTAAAGAGGTCA | UGT73C6 RT-qPCR |
| at2g36790-qRV | TCCACCAACACTCCTATCTTCTC | UGT73C6 RT-qPCR |
| at2g03760-qFW | GGTCACCAATCCACACCTTC | SOT12 RT-qPCR |
| at2g03760-qRV | CGAAATCTGGGGACTCGTAG | SOT12 RT-qPCR |
| at1g05680-qFW | GAATCGTCCTCATACCCGAAT | UGT74E2 RT-qPCR |
| at1g05680-qRV | GCTTTGGACCCATTTCAACA | UGT74E2 RT-qPCR |
| at2g04040-qFW | CATCAGCTGCAATGATTTGTC | DTX1 RT-qPCR |
| at2g04040-qRV | GAACAGAGGTCTCGAGTTTCG | DTX1 RT-qPCR |
| at2g21640-qFW | AAAAGAAAGAAGAAGCGAAGAAGA | UPOX RT-qPCR |
| at2g21640-qRV | CTTGGTATCCACCACGGTTT | UPOX RT-qPCR |
| SALK_030702_LP | CTTTTCCAAAAATGAAGGCG | T-DNA genotyping |
| SALK_030702_RP | TGAGCATCTTGAGTCCGACG | T-DNA genotyping |
| SALK_LB | ATTTTGCCGATTTCGGAAC | T-DNA genotyping |
| AttB4_P-UPOX_FW | ATAGAAAAGTTGACAACATTGATCATACGAGATCAAAAAGG | Y1H promoter cloning |
| AttB1r_P-UPOX_RV | TGTACAAACTTGACGCTGAAAACAGAAAGAAATCTCATGAA | Y1H promoter cloning |
| AttB4_P-UGT74E2_FW | GGGGACAACTTTGTATAGAAAAGTTGGATTTCACCCATGATATACTG | Y1H promoter cloning |
| AttB1r_P-UGT74E2_RV | GGGGACTGCTTTTTTGTACAAACTTGTTTCTCCTTCTTTTTAATCTTGT | Y1H promoter cloning |
| AttB4_P-AOX1a_FW | ATAGAAAAGTTGATCTGAAGAGCTTCTAGC | Y1H promoter cloning |
| AttB1r_P-AOX1a _RV | TGTACAAACTTGTGTTTCAAATCGGAAAAAGTG | Y1H promoter cloning |
